# Supplementary material for: A simplified method for evaluating swallowing ability and estimating malnutrition risk: A pilot study in older adults
Source: PLoS One. 2022 Feb 16;17(2):e0263896. doi: 10.1371/journal.pone.0263896 (PMC8849596; doi:10.1371/journal.pone.0263896)
Supplement: S1 File — (PDF) [file pone.0263896.s001.pdf]

Supporting Information

S1 File. Original nine common signs and symptoms of dysphagia

(Adopted from Walker et al (1990) and Nawaz S and Tulunay-Ugur OE (2018))

1) Having problems or difficulty swallowing certain food or liquids, or could not swallow at all.
2) Coughing or choking when eating or drinking.
3) Aspirations with liquids or solid food occurs.
4) A sensation that food got stuck in the throat or chest.
5) Painful swallowing.
6) Unintentional weight loss.
7) Having to wash down food with liquids.
8) Increased time needed to be able to complete meals.
9) Increased mucus in the throat.

Note: Items 1–4 were included in the final simplified swallowing questionnaire.

References:

- Walker HK, Hall WD, Hurst JW, editors. Chapter 82 - Dysphagia. Clinical Methods: The History, Physical, and Laboratory Examinations. 3rd edition Boston: Butterworths; 1990.
- Nawaz S, Tulunay-Ugur OE. Dysphagia in the older oatient. Otolaryngol Clin North Am 2018;51(4):769-777.
