# Supplementary material for: A simplified method for evaluating swallowing ability and estimating malnutrition risk: A pilot study in older adults
Source: PLoS One. 2022 Feb 16;17(2):e0263896. doi: 10.1371/journal.pone.0263896 (PMC8849596; doi:10.1371/journal.pone.0263896)
Supplement: S2 File — (PDF) [file pone.0263896.s002.pdf]

Supporting Information

S2 File. Questionnaire for data collection (Thai and English versions), Thai-version of Simplified Swallowing Questionnaire (T-SSQ), Thai-version of Mini-Mental State Evaluation (MMSE) (MMSE-Thai), and Thai-Mini Nutritional Assessment (MNA-Thai)

Questionnaire (Original Thai version)

แบบสอบถามและสัมภาษณ์

ผู้ให้ข้อมูล: [1] เจ้าตัวเอง [2] ผู้อื่น (โปรดระบุ): _____________

ข้อมูลทางสังคม

A. **เพศ** : [1] ชาย [2] หญิง

B. **อายุ**: _ _ _ ปี

C. **สถานะการสมรส**: [1] โสด [2] สมรส [3] แยกกันอยู่ [4] หย่าร้าง [5] ม่าย

D. **ระดับการศึกษาสูงสุด**: [1] ไม่ได้ศึกษา [2] ประถมหรือต่ำกว่า [3] มัธยม/เทียบเท่า (ปวช)
[4] ประกาศนียบัตรชั้นสูง หรืออนุปริญญา (ปวส) [5] ปริญญาตรีหรือสูงกว่า

สุขภาพร่างกาย

A. Clinical Frailty Scale (CFS): [1] very fit [2] well [3] managing well [4] vulnerable
[5] mildly frail [6] moderately frail [7] Severely frail
[8] Very Severely frail [9] Terminally Ill

B. **น้ำหนัก** _ _ _ กก. **ส่วนสูง** _ _ _ **ซม.**

C. **สภาวะโภชนาการ**: **คะแนนะ** MNA = _ _ _

D. **ภาวะการรับรู้**: **คะแนน** MMSE = _ _ _

สภาพช่องปากและฟันปลอม

A. **จำนวนฟันธรรมชาติที่เหลือ (ซี่)** _ _

B. **จำนวนคู่สบของฟันธรรมชาติ**

**ซี่ฟัน**	**ด้านซ้าย**	**ด้านขวา**
Premolar		
Molars		

C. **ขนิดฟันปลอมที่ใส่**: [1] ไม่มีฟันปลอม [2] ฟันปลอมบางส่วนถอดได้ (ฐานอะคริลิก หรือโลหะ)
[3] ฟันปลอมทั้งปากถอดได้

Questionnaire (English translation)

Respondent: [1] Older adults themselves [2] Others (please indicated): ___________

Sociodemographic

A. **Sex** : [1] male [2] female

B. **Age**: _ _ _ years:

C. **Marital status:** [1] single [2] married [3] separated
[4] divorced [5] widowed

D. **Highest level of education:** [1] uneducated [2] primary or lower
[3] secondary school or technical equivalent [4] post high school diploma / certificate
[5] bachelor or higher university degree

Health-related status

A. **Clinical Frailty Scale (CFS):** [1] very fit [2] well [3] managing well [4] vulnerable
[5] mildly frail [6] moderately frail [7] Severely frail
[8] Very Severely frail [9] Terminally Ill

A. weight _ _ _ kg Height _ _ _ cm

B. Nutritional status: MNA score = _ _ _

C. Cognitive function: MMSE score = _ _ _

Oral status

A. Number of remaining natural teeth _ _

B. Number of posterior occluding pair (natural teeth)

Teeth	Left side	Right side
Premolar		
Molars		

C. **Current denture type:** [1] No denture [2] Partial Dentures (Acrylic-based or Metal-based)
[3] Complete Denture

Thai-version of Simplified Swallowing Questionnaire (T-SSQ)

แบบสอบถามอย่างง่ายเกี่ยวกับปัญหาการกลืน

คำถาม: ในช่วง 1 เดือนที่ผ่านมา ท่านมีปัญหา....อย่างน้อย 1 ครั้งต่อสัปดาห์ใช่หรือไม่

ปัญหา	มีปัญหา	ไม่มีปัญหา
1) มีปัญหาหรือรู้สึกลำบากในการกลืนอาหารหรือน้ำ หรือไม่สามารถกลืนได้เลย		
2) มีอาการไอ สำลัก ขณะกินอาหารหรือดื่มน้ำ		
3) สูดสำลักน้ำหรืออาหารเข้าทางเดินหายใจ		
4) รู้สึกว่ามีอาหารติดในลำคอหรือทรวงอก		

Thai-version of Mini-Mental State Evaluation (MMSE) (MMSE-Thai 2002)

แบบทดสอบสภาพสมองเบื้องต้นฉบับภาษาไทย

ระดับการศึกษา ◌ ไม่ได้เรียน ◌ ประถมศึกษา ◌ มัธยมศึกษา ◌ อนุปริญญา/ปริญญาตรีขึ้นไป

***ในกรณีที่ผู้ถูกถามอ่านไม่ออก เขียนไม่ได้ไม่ต้องทำข้อ 4,9,10

	บันทึกคำตอบทุกครั้ง (ทั้งคำตอบที่ถูกและผิด)	คะแนน
1. Orientation for Time (5 คะแนนข้อละ 1 คะแนน)		
1.1 วันนี้ วันที่เท่าไร		
1.2 วันนี้ วันอะไร		
1.3 เดือนนี้ เดือนอะไร		
1.4 ปีนี้ ปีอะไร		
1.5 ฤดูนี้ ฤดูอะไร		
2.Orientation for Place (5 คะแนน) (ให้เลือกทำข้อใดข้อหนึ่ง) (ตอบถูกข้อละ 1 คะแนน)		
2.1 สถานที่ตรงนี้ เรียกว่าอะไร และชื่อ/บ้านเลขที่อะไร		
2.2 ขณะนี้ อยู่ที่ชั้นเท่าไรของตัวอาคาร		
2.3 ที่นี่อยู่ในอำเภอ – เขตอะไร		
2.4 ที่นี่จังหวัดอะไร		
2.5 ที่นี่ภาคอะไร		
3. Registration (3 คะแนน)		
ต่อไปนี้เป็นการทดสอบความจำ ผม(ดิฉัน) จะบอกชื่อของสามอย่าง คุณ(ตา,ยาย,...) ตั้งใจฟังให้ดีนะ เพราะจะบอกเพียงครั้งเดียว ไม่มีการบอกซ้ำอีก เมื่อผม(ดิฉัน) พูดจบ ให้คุณ(ตา,ยาย,...) พูดทบทวนตามที่ได้ยินให้ครบทั้งสามชื่อ แล้วพยายามจำไว้ให้ดี เดี๋ยวผม (ดิฉัน) จะถามซ้ำ *การบอกชื่อแต่ละคำให้ห่างกันประมาณ 1 วินาที ต้องไม่ช้าหรือเร็วเกินไป (ตอบถูก 1 คำ ได้ 1 คะแนน)		
( ) ดอกไม้ ( ) แม่น้ำ ( ) รถไฟ		
( ) ต้นไม้ ( ) ทะเล ( ) รถยนต์ (กรณีทำซ้ำใน 2เดือน)		

	บันทึกคำตอบทุกครั้ง (ทั้งคำตอบที่ถูกและผิด)	คะแนน
4. Attention /Calculation (5 คะแนน) ให้เลือกทำข้อใดข้อหนึ่ง		
ข้อนี้เป็นการคิดเลขในใจ เพื่อทดสอบสมาธิ คุณ (ตา,ยาย...) คิดเลขในใจเป็นไหม? * ถ้าตอบคิดเป็นให้ตอบข้อ 4.1 * ถ้าตอบคิดไม่เป็นหรือไม่ตอบ ให้ตอบข้อ 4.2		
4.1 "ข้อนี้คิดในใจ เอา 100 ตั้ง ลบออกทีละ 7 ไปเรื่อยๆ ได้ผลลัพธ์เท่าไร บอกมา"<br>บันทึกตัวเลขไว้ทุกครั้ง (ทั้งคำตอบที่ถูกหรือผิด) ทำทั้งหมด 5 ครั้ง ถ้าลบได้ 1,2 หรือ 3 แล้วตอบไม่ได้ ให้คิดคะแนนเท่าที่ทำได้ โดยไม่ต้องย้ายไปทำข้อ 4.2		
4.2 "ผม (ดิฉัน) สะกดคำว่ามะนาว ให้คุณ (ตา,ยาย,...) ฟัง แล้วให้คุณ (ตา,ยาย,...)สะกดถอยหลังจากพยัญชนะตัวหลังไปตัวแรก" คำว่า มะนาว สะกดว่า มอม้า-สระอะ-นอหนู-สระอา-วอแหวน ไหนคุณ (ตา,ยาย,...) สะกดถอยหลังให้ฟังซิ	....<br>ว า น ะ ม	
5. Recall (3 **คะแนน**)<br>"เมื่อสักครู่ที่ให้จำของ 3 อย่า จำได้ไหม มีอะไรบ้าง"<br>(ตอบถูก 1 คำ ได้ 1 คะแนน)		
( ) ดอกไม้ ( ) แม่น้ำ ( ) รถไฟ		
( ) ต้นไม้ ( ) ทะเล ( ) รถยนต์		
6. Naming (2 คะแนน)		
6.1 ยื่นดินสอให้ผู้สูงอายุแล้วถามว่า"ของสิ่งนี้เรียกว่าอะไร"		
6.2 ชี้นาฬิกาข้อมือให้ผู้สูงอายุดูแล้วถามว่า "ของสิ่งนี้เรียกว่าอะไร"		
7. Repetition (พูดตามได้ถูกต้องได้ 1 คะแนน)		
"ตั้งใจฟังผม (ดิฉัน) นะ เมื่อผม (ดิฉัน) ให้คุณ (ตา,ยาย,...)พูดตามผม (ดิฉัน) จะบอกเพียงเที่ยวเดียว "<br>**"ใคร ใคร่ ขาย ไก่ ไข่"**		

	บันทึกคำตอบทุกครั้ง<br>(ทั้งคำตอบที่ถูกและผิด)	คะแนน
8. Verbal command (3 คะแนน)		
“ฟังดีดีนะ เดี๋ยวผม (ดิฉัน)จะส่งกระดาษให้ แล้วคุณ (ตา,ยาย,..)รับด้วยมือขวา พับครึ่งแล้ววางที่. (พื้น,โต๊ะ,เตียง)<br>ผู้ทดสอบแสดงกระดาษเปล่า ขนาดประมาณ เอ – 4 ไม่มีรอยพับให้ผู้สูงอายุ ( ) รับด้วยมือขวา ( ) พับครึ่ง ( ) แล้ววางที่.... (พื้น,โต๊ะ,เตียง)		
9. Written command (1คะแนน)		
ต่อไปนี้เป็นคำสั่งที่เขียนเป็นตัวหนังสือ ต้องการให้คุณ (ตา,ยาย,..) อ่านแล้วทำตาม คุณ(ตา,ยาย,..) จะอ่านออกเสียงหรือในใจก็ได้ ผู้ทดสอบแสดงกระดาษให้ผู้สูงอายุดู<br>**หลับตา**<br>( ) หลับตาได้		
10. Writing (1 คะแนน)		
ข้อความนี้เป็นคำสั่งให้คุณ (ตา,ยาย,...) เขียนข้อความอะไรก็ได้ ที่อ่านแล้วรู้เรื่อง หรือมีความหมายมา 1 ประโยค	......................................<br>......................................<br>......................................	
11. Visuo-construction (1 คะแนน)		
ข้อนี้เป็นคำสั่ง “จงวาดให้เหมือนภาพตัวอย่าง”ในที่ว่างด้านข้างของภาพตัวอย่าง<br>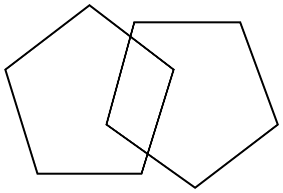<br>รูปห้าเหลี่ยมต้องมีมุม 5 มุม ตามภาพตัวอย่าง การตัดกันต้องเกิดรูปสี่เหลี่ยมด้ายในทำตามได้ทั้งหมดจึงจะได้คะแนน 1 คะแนน		
	**คะแนนรวม**	

**จุดตัด Cut-off สำหรับคะแนนที่สงสัยภาวะสมองเสื่อม (Congenital impaired) ตามระดับการศึกษา**	**คะแนน**	
	จุดตัด	เต็ม
- ผู้สูงอายุปกติ ไม่ได้เรียนหนังสือ (อ่านไม่ออกเขียนไม่ได้)	≤ 14	23<br>(ต้องไม่ทำข้อ 4,9,10)
- ผู้สูงอายุปกติ เรียนระดับประถมศึกษา	≤ 17	30
- ผู้สูงอายุปกติ เรียนระดับสูงกว่าประถมศึกษา	≤ 22	30

Reference: Thai Cognitive Test Development Committee 1999. Mini-Mental State Examination-Thai 2002 Bangkok: Institute of Geriatric Medicine, Department of Medical Services, Ministry of Public Health, Thailand. (2002). ISBN 974-9593-33-2

หลับตา

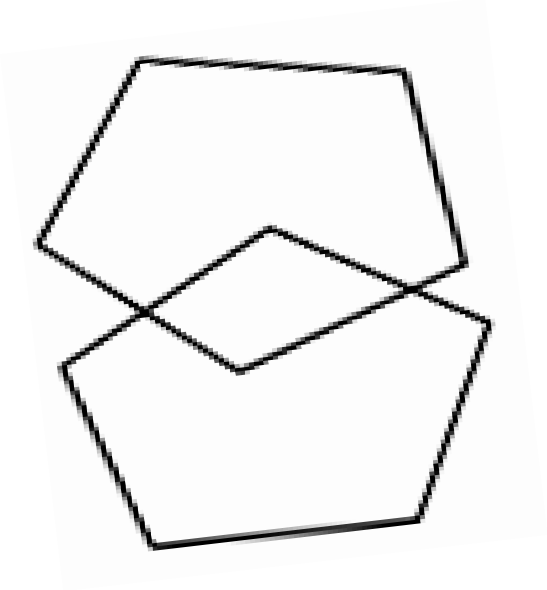

แบบประเมินภาวะโภชนาการ (Thai-Mini Nutritional Assessment; MNA®)

**ส่วนที่ 1 การคัดกรองภาวะโภชนาการเบื้องต้น**	
**คำถามคัดกรอง**	**คะแนน**
1. ในช่วง 3 เดือนที่ผ่านมามีการรับประทานอาหารได้น้อยลงเนื่องจากความอยากอาหารลดลง มีปัญหาการย่อย การเคี้ยว หรือปัญหาการกลืนหรือไม่<br>0 = รับประทานอาหารน้อยลงอย่างมาก 1 = รับประทานอาหารน้อยลงปานกลาง<br>2 = การรับประทานอาหารไม่เปลี่ยนแปลง	
2. ในช่วง 3 เดือนที่ผ่านมาน้ำหนักลดลงหรือไม่<br>0 = น้ำหนักลดลงมากกว่า 3 กิโลกรัม 1 = ไม่ทราบ<br>2 = น้ำหนักลดลงระหว่าง 1-3 กิโลกรัม 3 = น้ำหนักเท่าเดิม<br>3. สามารถเคลื่อนไหวได้เองหรือไม่<br>0 = นอนบนเตียงหรือต้องอาศัยรถเข็นตลอดเวลา<br>1 = ลุกจากเตียงหรือรถเข็นได้บ้าง แต่ไม่สามารถไปข้างนอกได้เอง<br>2 = เดินและเคลื่อนไหวได้ตามปกติ	
4. ในเดือนที่ผ่านมามีความเครียดรุนแรงหรือป่วยเฉียบพลันหรือไม่<br>0 = มี 2 = ไม่มี	
5. มีปัญหาทางประสาท (Neuropsychological Problems) หรือไม่<br>0 = ความจำเสื่อม หรือหดหู่อย่างรุนแรง 1 = ความจำเสื่อมเล็กน้อย<br>2 = ไม่มีปัญหาทางจิตประสาท	
6. ดัชนีมวลกาย [(BMI) = น้ำหนัก (กก.)/ [ส่วนสูง (ม.^{2})]<br>0 = BMI น้อยกว่า 19 1 = BMI ตั้งแต่ 19 แต่น้อยกว่า 21<br>2 = BMI ตั้งแต่ 21 แต่น้อยกว่า 23 3 = BMI ตั้งแต่ 23 ขึ้นไป	

**ส่วนที่ 2 การประเมินภาวะโภชนาการ**	
**คำถามคัดกรอง**	**คะแนน**
7. คุณอาศัยอยู่ลำพังได้โดยไม่ต้องพึ่งพาผู้อื่น (ไม่ได้อยู่ในสถานพักคนชรา หรือ โรงพยาบาล) ใช่หรือไม่ 0 = ไม่ใช่ 1 = ใช่	
8. คุณรับประทานยามากกว่า 3 ชนิดต่อวัน ใช่หรือไม่ 0 = ใช่ 1 = ไม่ใช่	
9. คุณมีแผลกดทับหรือแผลที่ผิวหนังอื่นหรือไม่ 0 = ใช่ 1 = ไม่ใช่	
10. คุณรับประทานอาหารได้ครบ 5 หมู่ วันละกี่มื้อ 0 = 1 มื้อ 1 = 2 มื้อ 2 = 3 มื้อ	
11. ในแต่ละวันคุณรับประทานโปรตีนบ้างหรือไม่<br>*รับประทานนมหรือผลิตภัณฑ์นม (เช่น ชีส โยเกิร์ต) อย่างน้อยวันละ 1 หน่วยบริโภค ใช่ หรือไม่<br>*รับประทานถั่วเม็ดแห้งหรือไข่ 2 ครั้งต่อสัปดาห์ใช่หรือไม่<br>*รับประทานเนื้อสัตว์ปลา หรือสัตว์ปีก ทุกวัน ใช่หรือไม่<br>0.0 ถ้าตอบไม่ใช่ทุกข้อหรือใช่เพียงข้อเดียว 0.5 ถ้าตอบใช่ 2 ข้อ 1.0 ถ้าตอบใช่ 3 ข้อ	
12. คุณรับประทานผักหรือผลไม้อย่างน้อย 2 มื้อต่อวัน ใช่หรือไม่ 0 = ไม่ใช่ 1 = ใช่	
13. คุณดื่มน้ำหรือเครื่องดื่มต่างๆ เช่น น้ำ น้ำผลไม้กาแฟ ชา นม ปริมาณวันละเท่าไร<br>0.0 = น้อยกว่า 3 แก้ว/วัน 0.5 = 3-5 แก้ว/วัน 1.0 = มากกว่า 5 แก้ว/วัน	
14. ในการรับประทานอาหาร คุณสามารถช่วยเหลือตนเองได้หรือไม่<br>0 = ไม่สามารถรับประทานอาหารได้ด้วยตนเอง<br>1 = รับประทานอาหารเองได้แต่ค่อนข้างลำบาก<br>2 = รับประทานอาหารได้โดยไม่มีปัญหา	
15. คุณคิดว่าตนเองมีภาวะโภชนาการเป็นอย่างไร<br>0 = เห็นว่าตนเองน่าจะมีภาวะทุพโภชนาการ<br>1 = ไม่แน่ใจว่ามีภาวะทุพโภชนาการ<br>2 = เห็นว่าตนเองไม่มีภาวะทุพโภชนาการ	

ส่วนที่ 2 การประเมินภาวะโภชนาการ	
**คำถามคัดกรอง**	**คะแนน**
16. เมื่อเปรียบเทียบกับบุคคลอื่นในวัยเดียวกัน คุณคิดว่าสุขภาพของตนเองเป็นอย่างไร<br>0.0 = ไม่ดีเท่ากับคนอื่น 0.5 = ไม่ทราบ<br>1.0 = ดีเท่ากับคนอื่น 2.0 = ดีกว่าคนอื่น<br>17. เส้นรอบวงแขน (Mid Arm Circumference: MAC)<br>0.0 = MAC น้อยกว่า 21 ซม. 0.5 = MAC 21 ถึง 22 ซม.<br>1.0 = MAC มากกว่า 22 ซม.<br>18. เส้นรอบวงน่อง (Calf Circumference: CC)<br>0.0 = CC น้อยกว่า 31 ซม. 1.0 = CC ตั้งแต่ 31 ซม. ขึ้นไป รวมคะแนน	
**คะแนนรวม**	
**การแปลผล** 24-30 คะแนน มีภาวะโภชนาการปกติ<br>17-23.5 คะแนน มีความเสี่ยงต่อภาวะทุพโภชนาการ<br>น้อยกว่า 17 คะแนน มีภาวะทุพโภชนาการ	

Reference: Handbook for standard and operation of qualified elderly clinic. In.: Institute of Geriatric Medicine, Department of Medical Service, Ministry of Public Health (2013).
