# Supplementary material for: A simplified method for evaluating swallowing ability and estimating malnutrition risk: A pilot study in older adults
Source: PLoS One. 2022 Feb 16;17(2):e0263896. doi: 10.1371/journal.pone.0263896 (PMC8849596; doi:10.1371/journal.pone.0263896)
Supplement: Attachment — Submitted filename: PLOS ONE revised.pdf [file pone.0263896.s005.pdf]

PLOS ONE

A simplified method for evaluating swallowing ability and estimating malnutrition risk in older adults

--Manuscript Draft--

**Manuscript Number:**	PONE-D-21-21718
**Article Type:**	Research Article
**Full Title:**	A simplified method for evaluating swallowing ability and estimating malnutrition risk in older adults
**Short Title:**	Swallowing ability and malnutrition risk
**Corresponding Author:**	Orapin Komin, Ph.D.<br>Chulalongkorn University Faculty Of Dentistry<br>Bangkok, Thailand THAILAND
**Keywords:**	Nutritional assessment; Oral function; Swallowing function; Tongue function; Tongue pressure.
**Abstract:**	Objectives: To evaluate the association between malnutrition risk and swallowing ability, determined objectively by tongue strength and subjectively by a newly-developed 4-item questionnaire. Sensitivity analysis was also performed to determine which swallowing indices better estimate malnutrition in older adults.<br>Methods: This cross-sectional study included 60 older adults. The dependent variable was nutritional status evaluated using the Mini-Nutritional Assessment. The independent variables were subjective and objective swallowing ability, evaluated using a 4-item questionnaire of swallowing problems and tongue strength, respectively. Adjusting for covariates, the associations between the two swallowing indices and malnutrition risk were determined using multivariable regression analyses. A cut-off value for low tongue strength was determined using a receiver operating characteristic (ROC) curve, and sensitivity analysis between the swallowing indices and malnutrition risk was performed.<br>Results: Convergent validity of subjective index was revealed by its significant association with objective tongue strength. Based on the highest area under the ROC curve, an 18-kPa cut-off value was chosen to classify low tongue strength. Having a swallowing problem and low tongue strength was significantly associated with malnutrition risk. PPV values of subjective swallowing index was about 1.8-fold higher than objective tongue strength.<br>Conclusions: Self-reported swallowing problems can be used as a subjective index for evaluating swallowing ability in older adults. Subjective swallowing problems and objective tongue strength were associated with malnutrition risk. However, the subjective index better estimated malnutrition risk than the objective index.
**Order of Authors:**	Nareudee Limpuangthip
	Orapin Komin, Ph.D.
	Teerawut Tatiyapongpaiboon
**Additional Information:**	
**Question**	**Response**
**Financial Disclosure**<br><br>Enter a financial disclosure statement that describes the sources of funding for the work included in this submission. Review the submission guidelines for detailed requirements. View published research articles from *PLOS ONE* for specific examples.	Yes. This research is funded by Chulalongkorn University, Grant number CU_GR_63_11_32_04. The funders had no role in study design, data collection and analysis, decision to publish, or preparation of the manuscript.

Powered by Editorial Manager® and ProduXion Manager® from Aries Systems Corporation

This statement is required for submission and **will appear in the published article** if the submission is accepted. Please make sure it is accurate.<br><br>**Unfunded studies**<br>Enter: *The author(s) received no specific funding for this work.*<br><br>**Funded studies**<br>Enter a statement with the following details:<br>• Initials of the authors who received each award<br>• Grant numbers awarded to each author<br>• The full name of each funder<br>• URL of each funder website<br>• Did the sponsors or funders play any role in the study design, data collection and analysis, decision to publish, or preparation of the manuscript?<br>• **NO** - Include this sentence at the end of your statement: *The funders had no role in study design, data collection and analysis, decision to publish, or preparation of the manuscript.*<br>• **YES** - Specify the role(s) played.<br><br>* typeset	
**Competing Interests**<br><br>Use the instructions below to enter a competing interest statement for this submission. On behalf of all authors, disclose any competing interests that could be perceived to bias this work—acknowledging all financial support and any other relevant financial or non-financial competing interests.<br><br>This statement is required for submission and **will appear in the published article** if the submission is accepted. Please make sure it is accurate and that any funding sources listed in your Funding Information later in the submission form are also declared in your Financial Disclosure statement.<br><br>View published research articles from *PLOS ONE* for specific examples.	The authors have declared that no competing interests exist.

**NO authors have competing interests**<br><br>Enter: *The authors have declared that no competing interests exist.*<br><br>**Authors with competing interests**<br><br>Enter competing interest details beginning with this statement:<br><br>*I have read the journal's policy and the authors of this manuscript have the following competing interests: [insert competing interests here]*<br><br>* typeset	
**Ethics Statement**<br><br>Enter an ethics statement for this submission. This statement is required if the study involved:<br><br>• Human participants<br>• Human specimens or tissue<br>• Vertebrate animals or cephalopods<br>• Vertebrate embryos or tissues<br>• Field research<br><br>Write "N/A" if the submission does not require an ethics statement.<br><br>General guidance is provided below. Consult the submission guidelines for detailed instructions. **Make sure that all information entered here is included in the Methods section of the manuscript.**	The study protocol was approved by the Ethics Committee of the Faculty of Dentistry, Chulalongkorn University (HREC-DCU 2018-112).

**Format for specific study types**<br><br>**Human Subject Research (involving human participants and/or tissue)**<br>• Give the name of the institutional review board or ethics committee that approved the study<br>• Include the approval number and/or a statement indicating approval of this research<br>• Indicate the form of consent obtained (written/oral) or the reason that consent was not obtained (e.g. the data were analyzed anonymously)<br><br>**Animal Research (involving vertebrate animals, embryos or tissues)**<br>• Provide the name of the Institutional Animal Care and Use Committee (IACUC) or other relevant ethics board that reviewed the study protocol, and indicate whether they approved this research or granted a formal waiver of ethical approval<br>• Include an approval number if one was obtained<br>• If the study involved *non-human primates*, add *additional details* about animal welfare and steps taken to ameliorate suffering<br>• If anesthesia, euthanasia, or any kind of animal sacrifice is part of the study, include briefly which substances and/or methods were applied<br><br>**Field Research**<br><br>Include the following details if this study involves the collection of plant, animal, or other materials from a natural setting:<br>• Field permit number<br>• Name of the institution or relevant body that granted permission	
**Data Availability**<br><br>Authors are required to make all data underlying the findings described fully available, without restriction, and from the time of publication. PLOS allows rare exceptions to address legal and ethical concerns. See the PLOS Data Policy and FAQ for detailed information.	No - some restrictions will apply

Powered by Editorial Manager® and ProduXion Manager® from Aries Systems Corporation

A Data Availability Statement describing where the data can be found is required at submission. Your answers to this question constitute the Data Availability Statement and **will be published in the article**, if accepted.<br><br>**Important:** Stating 'data available on request from the author' is not sufficient. If your data are only available upon request, select 'No' for the first question and explain your exceptional situation in the text box.<br><br>Do the authors confirm that all data underlying the findings described in their manuscript are fully available without restriction?	
**Describe where the data may be found in full sentences. If you are copying our sample text, replace any instances of XXX with the appropriate details.**<br><br>• If the data are **held or will be held in a public repository**, include URLs, accession numbers or DOIs. If this information will only be available after acceptance, indicate this by ticking the box below. For example: *All XXX files are available from the XXX database (accession number(s) XXX, XXX.).*<br>• If the data are all contained **within the manuscript and/or Supporting Information files**, enter the following: *All relevant data are within the manuscript and its Supporting Information files.*<br>• If neither of these applies but you are able to provide **details of access elsewhere**, with or without limitations, please do so. For example:<br><br>*Data cannot be shared publicly because of [XXX]. Data are available from the XXX Institutional Data Access / Ethics Committee (contact via XXX) for researchers who meet the criteria for access to confidential data.*<br><br>*The data underlying the results presented in the study are available from (include the name of the third party*	All relevant data are within the manuscript and its Supporting Information files.

and contact information or URL).<br>• This text is appropriate if the data are owned by a third party and authors do not have permission to share the data.<br><br>* typeset	
Additional data availability information:	Tick here if the URLs/accession numbers/DOIs will be available only after acceptance of the manuscript for publication so that we can ensure their inclusion before publication.

Powered by Editorial Manager® and ProduXion Manager® from Aries Systems Corporation

Manuscript Click here to access/download;Manuscript;Manu_Swallow_PlosOne.docx

Title page

A simplified method for evaluating swallowing ability and estimating malnutrition risk in older adults

Short title: Swallowing ability and malnutrition risk

Nareudee Limpuangthip[1¶], Orapin Komin[1¶], Teerawut Tatiyapongpaiboon[2]

[1]Department of Prosthodontics, Faculty of Dentistry, Chulalongkorn University, Bangkok, Thailand.

[2]Thungyai Hospital, Nakhon Si Thammarat, Thailand

*** Corresponding author:**

E-mail: Orapin.geriatric@gmail.com

Abstract

Objectives: To evaluate the association between malnutrition risk and swallowing ability, determined objectively by tongue strength and subjectively by a newly-developed 4-item questionnaire. Sensitivity analysis was also performed to determine which swallowing indices better estimate malnutrition in older adults.

Methods: This cross-sectional study included 60 older adults. The dependent variable was nutritional status evaluated using the Mini-Nutritional Assessment. The independent variables were subjective and objective swallowing ability, evaluated using a 4-item questionnaire of swallowing problems and tongue strength, respectively. Adjusting for covariates, the associations between the two swallowing indices and malnutrition risk were determined using multivariable regression analyses. A cut-off value for low tongue strength was determined using a receiver operating characteristic (ROC) curve, and sensitivity analysis between the swallowing indices and malnutrition risk was performed.

Results: Convergent validity of subjective index was revealed by its significant association with objective tongue strength. Based on the highest area under the ROC curve, an 18-kPa cut-off value was chosen to classify low tongue strength. Having a swallowing problem and low tongue strength was significantly associated with malnutrition risk. PPV values of subjective swallowing index was about 1.8-fold higher than objective tongue strength.

Conclusions: Self-reported swallowing problems can be used as a subjective index for evaluating swallowing ability in older adults. Subjective swallowing problems and objective tongue strength were associated with malnutrition risk. However, the subjective index better estimated malnutrition risk than the objective index.

Keywords: Nutritional assessment, Oral function, Swallowing function, Tongue function, Tongue pressure.

Introduction

Oral and general health functionally decline as people age [1]. Gradually declined oral function can lead to oral frailty followed by oral hypofunction. However, they can recover to the healthy stage by early detection and proper dental treatment. Oral health becomes oral frailty when a person has decreased occluding teeth, increased unchewable foods, or slight choking/spillage while eating. Moreover, oral hypofunction is diagnosed when 3 out of 7 oral signs or symptoms are present: oral uncleanness and dryness, reduced occlusal force, reduced chewing function, reduced tongue and lip motor function, as well as reduced tongue pressure and swallowing function [2]. Because eating and swallowing ability plays a major role in oral function, a decline in swallowing ability contributes to malnutrition [3, 4]. Malnutrition increases the risk of morbidity and mortality, and negatively affect the quality of life of older adults [5].

To prevent malnutrition in older adults, early detection of declined swallowing ability is necessary. Several objective and subjective indices have been used to evaluate swallowing ability in older adults. Tongue pressure measurement is commonly used to objectively evaluate swallowing ability, because tongue motor function plays an important role in mastication and swallowing [3, 6]. However, this method requires special instruments and time to perform. To evaluate swallowing ability subjectively, the 10-item Eating Assessment Tool (EAT-10) is commonly used because it is considered a reliable and validated questionnaire [2, 6, 7]. However, some studies reported the limitations of EAT-10 regarding its substantial floor effect,

several redundant items, and relatively low construct validity [8, 9]. Thus, an alternative simple screening method for evaluating swallowing ability should be proposed for the early detection of oral function when a patient is in the frailty or hypofunction stage.

The aim of the present study was to evaluate the association between malnutrition risk and swallowing ability, determined objectively by tongue strength and subjectively by a newly-developed 4-item questionnaire. In addition, sensitivity analysis was performed to determine which swallowing indices better estimate malnutrition in older adults.

Materials and Methods

Study design and participants

The present study was a cross-sectional design. The study protocol was approved by the Ethics Committee of the Faculty of Dentistry, Chulalongkorn University (HREC-DCU 2018-112). The participants and their guardians gave written informed consent prior to participating in the study.

The participants were recruited from patients who received dental treatment at the Geriatric and Special Patients Care Clinic, Faculty of Dentistry, Chulalongkorn University during 2017–2019. The exclusion criteria were patients who declined or were unable to perform a tongue pressure test due to severely declined functional or intellectual conditions, or currently had malnutrition. Based on these criteria, 61 older adults aged ≥ 60 years old participated in the study.

Power analysis of the sample size was calculated based on the null hypothesis of two independent proportions. The results indicated that the prevalence of malnutrition risk in participants who reported swallowing problem (n_1=7) and those who did not (n_2=54) were 0.71 and 0.11, respectively. Thus, a power of 95.2% was calculated at 5% type I error.

Dependent variables

Nutritional status was measured using the Thai-version of the Mini-Nutritional Assessment (MNA) with a score ranging from 0–30 [10]. The participants were categorized as having malnutrition risk when MNA score = 17–23.5, and being normal when the score ≥24. The MNA was used because it is a standardized and validated instrument in older adults [10].

Independent variables

Swallowing ability was evaluated subjectively by a newly-developed self-reported swallowing problems and objectively by tongue strength. In this study, the subjective swallowing index was a 4-item questionnaire, in which the items were adopted from common signs and symptoms reported by patients with swallowing problems [11]. Swallowing ability was evaluated by interviewing the participants with the assistance of their caregivers, if present. The participants were defined as having a swallowing problem when at least one of the following signs and symptoms was present at least once a week within the past month: 1) having problems swallowing certain food or liquids, or could not swallow at all, 2) coughing or choking when eating or drinking, 3) bringing food back up, sometimes through the nose, or 4) a sensation that food got stuck in the throat or chest. The inter-examiner reliability was determined using 15 patients at the first patients' evaluation visit. The intra-examiner reliability was evaluated by

reinterviewing these 15 patients one week later. The weighted Kappa scores for the inter- and intra-examiner reliability assessments were approximately 0.82 and 0.87, respectively.

The objective swallowing ability was evaluated through tongue pressure, measured using the JMS TPM-02 measurement device (JMS, Inc., Hiroshima, Japan), which consisted of a plastic catheter and a balloon [12-14]. The participants sat in an upright position. The balloon was inserted into their oral cavity and placed on the anterior part of the palate with their lips and jaw closed, while the plastic catheter was held at the midpoint of the central incisors. The participants raised their tongue and pressed the balloon against the hard palate as hard as possible, and the maximum tongue pressure (kPa, kilopascal) was read. This procedure was done in triplicate with 5 min resting intervals, and the tongue strength (kPa) was calculated from the average value of the three measurements. The objective tongue strength was used as a reference to assess the convergent validity of the proposed subjective swallowing index.

Covariates

Information regarding biological factors, oral- and health-related status was recorded. Biological factors were an individuals' age and sex. A dental professional evaluated oral status, comprising the number of remaining functional teeth (ranged from 0 to 28 teeth), number of posterior occluding pairs (ranged from 0 to 8 pairs), and type of denture worn. If more than one type of denture presented, it was classified as the type with a higher number of tooth loss.

Health-related status covered the participants physical and psychological conditions: dependency status and cognitive status, respectively. The clinical frailty scale (CFS) was used to categorize dependency status into independent, semi-dependent, and dependent [15]. Cognitive function was evaluated using the Thai-version of Mini-Mental State Evaluation (MMSE) [16].

With a score ranging from 0–30, the participants were considered as having mild cognitive impairment (MCI) when the score was below 18 and 22 when their educations was at least primary and above primary level, respectively.

Data analysis

Descriptive statistics was performed to determine the percentage (%) and mean ±standard deviation (s.d.). Univariate analyses of the associations between related variables and having a swallowing problem were analyzed using the chi-squared test, whereas its association with tongue strength and MNA score were analyzed using either one-way ANOVA or independent t-test. Variables with p-value < 0.10 were included in the multivariable analyses. Adjusting for covariates, multivariable logistic and linear regression were used to determine the factors associated with the subjective and objective swallowing indices, and their associations with malnutrition risk. A receiver operating characteristic (ROC) curve was plotted to determine the area under the curve (AUC) in the malnutrition risk models; the higher the AUC, the better the model was able to distinguish between the participants with and without malnutrition risk. To categorize the low and high tongue strength, a cut-off value that gave the highest AUC value was chosen. For the sensitivity analysis, the positive predictive value (PPV), negative predictive value (NPV), sensitivity, and specificity between malnutrition risk and the two swallowing ability indices were calculated. The data were analyzed using STATA version 13.0 (StataCorp LP) at a 5% significance level.

Results

The characteristics of the participants based on the subjective and objective swallowing indices, and malnutrition risk are shown in Table 1. The mean ±s.d. age of participants was 78.0 ±7.0 years old. Malnutrition risk was found in 18% of the participants, while the others were within normal limits. MCI was present in 90% of semi- and dependent older participants. Oral status was associated with the subjective and objective swallowing indices, and malnutrition risk. The convergent validity of the subjective swallowing problem was revealed by its significant association with objective tongue strength.

Table 1. Characteristics of the study participants.

Variables	Overall distribution: %	Swallowing ability index: Self-reported swallowing problem (Yes): %	Swallowing ability index: Maximum tongue pressure (kPa): mean (±s.d.)	Nutritional status: MNA score: mean (±s.d.)
Overall	***100.0***	***11.7***	***25.5 (±10.1)***	***26.0 (±3.0)***
Age (years): 60 – 69	15.0	11.1	34.5 (±8.7)	25.0 (±3.8)
70 – 79	40.0	4.2	26.8 (±9.3)*	26.5 (±2.1)
80 and above	45.0	18.5†	21.4 (±9.4)*	26.0 (±3.3)
Sex: Male	48.3	10.3	27.7 (±9.8)	26.9 (±2.1)
Female	51.7	12.9	23.5 (±10.1)†	25.2 (±3.4)
**Health-related status**				
Dependency status: Independent	71.7	4.6	27.8 (±9.8)	26.9 (±2.1)
Semi-dependent	18.3	27.3*	19.1 (±8.9)*	25.1 (±2.5)
Dependent	10.0	33.3*	21.2 (±8.8)*	21.1 (±3.9)*
MMSE score: Normal	78.3	6.2	26.5 (±10.4)	26.5 (±2.2)
Mild cognitive impairment	21.7	30.8*	22.1 (±8.6)†	24.1 (±4.4)
**Oral status**				
Natural teeth: 0	19.7	16.7	24.1 (±10.2)	26.0 (±3.6)
1 - 19	70.5	11.6	25.2 (±9.7)	25.9 (±3.0)
20 and above	9.8	0.0	30.1 (±9.9)	27.0 (±2.0)
Posterior occluding pairs: 0	76.7	13.0	24.5 (±10.2)	26.1 (±3.0)
1 - 3	13.3	12.5	32.4 (±10.7)	25.9 (±1.8)
4 and above	10.0	0.0	26.7 (±9.6)	25.4 (±4.5)
Denture type: No denture	3.3	0.0	30.7 (±1.8)	29.2 (±1.1)
Removable partial denture	45.0	7.4	27.3 (±8.8)	26.0 (±2.7)
Complete denture	51.7	16.1	23.6 (±11.1)	25.8 (±3.2)
**Swallowing ability**				
Having swallowing problem: No	11.7	-	26.7 (±9.6)	26.0 (±2.8)
Yes	88.3		16.8 (±10.6)*	23.0 (±2.7)*

*p <0.05, †p<0.10. N/A, not applicable due to multicollinearity with dependency status.

OR, odds ratio; β, beta-coefficient; CI, confidence interval; ref, reference.

There were significant associations between the subjective and objective swallowing indices and malnutrition risk after adjusting for potential covariates (Table 2). Because there was collinearity between dependency status and MCI, the MCI variable was not included in the multivariable regression models. Based on the ROC curve, 18 kPa was chosen as a cut-off value to categorize the participants into low and high tongue strength because it gave the highest AUC value when plotting the curve between tongue strength and malnutrition risk (Figure 1).

Table 2. The associations between swallowing indices and related variables.

Variables	Swallowing ability index		Malnutrition risk: adjusted OR (95% CI)	
	Self-reported swallowing problem (Yes): adjusted OR (95% CI)	Maximum tongue pressure (kPa): adjusted β (95% CI)	Model 1	Model 2
Age (years old)	1.05 (0.92, 1.18)	-0.65 (-1.00, -0.30)*	1 (ref)	0.98 (0.84, 1.14)
Sex (Female)	0.55 (0.07, 4.38)	-1.00 (-5.76, 3.76)	6.28 (0.44, 45.4)	3.23 (0.45, 23.4)
Dependency status: Independent	1 (ref)	1 (ref)	1 (ref)	1 (ref)
Semi-dependent	8.44 (0.89.30.1)†	-4.19 (-10.6, 2.28)	0.21 (0.01, 6.26)	0.80 (0.07, 8.65)
Dependent	13.4 (1.24, 39.4)*	-6.67 (-14.1, 1.18)†	22.3 (1.53, 53.3)*	40.6 (3.30, 85.3)*
**Swallowing ability measures**				
Self-reported swallowing problem (Yes)			35.5 (3.49, 75.5)*	-
Maximum tongue pressure (<18 kPa)			-	0.11 (0.01, 0.71)*
AUC (%)			***88.3***	***74.9***

*p <0.05, †p<0.10. OR, odds ratio; β, beta-coefficient; CI, confidence interval; ref, reference; AUC, Area under the Received Operating Curve (ROC).

Figure 1. Receiver Operating Curves (ROC) and % Area under the ROC curve (AUC) of the association between malnutrition risk and swallowing indices after adjusting for covariates. 1a) Subjective swallowing problem, and 1b) Objective tongue strength (18-kPa cut-off value).

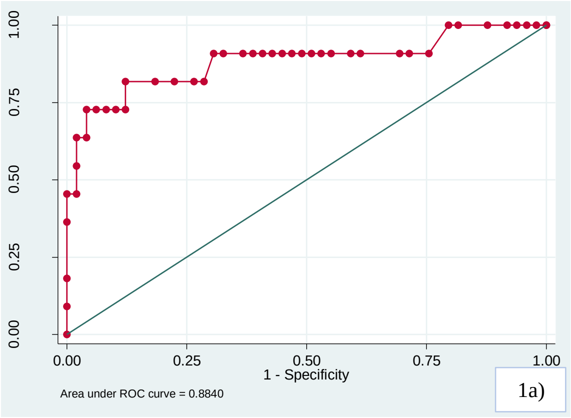

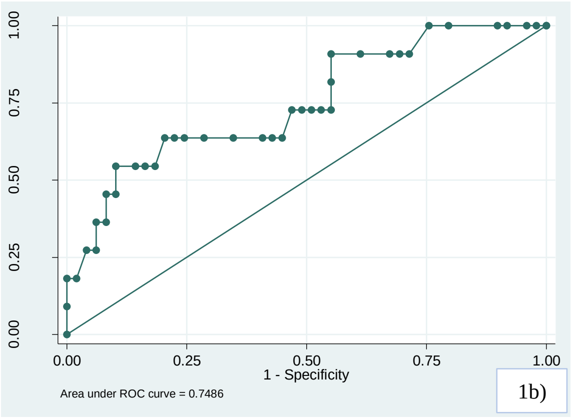

Estimates of the PPV, NPV, sensitivity, and specificity are presented in Table 3. The sensitivity value indicated that 45.5% and 36.4% of older adults having malnutrition risk would have a swallowing problem and lower tongue strength, respectively. The PPV values indicated that the participants with a swallowing problem were 1.5–2 folds more likely to have malnutrition risk than those who had lower tongue strength.

Table 3. PPV, NPV, sensitivity, and specificity (%) between swallowing indices and malnutrition risk.

Impaired swallowing ability	Malnutrition risk: (%)			
	PPV	NPV	Sensitivity	Specificity
Self-reported swallowing problem (Yes)	71.4	87.2	45.5	96.0
Low tongue strength: (< 18 kPa)	40.0	86.0	36.4	87.8

Discussion

The present study developed a 4-item questionnaire as a subjective index to evaluate the swallowing ability in older adults. The convergent validity of the subjective swallowing index was verified using objective tongue pressure as a reference. The findings revealed the association between the subjective and objective swallowing indices and malnutrition risk. Sensitivity analysis demonstrated that the ability of the subjective swallowing index in estimating malnutrition risk was better than objective tongue strength. In this study, the standard EAT-10 was not used as a subjective swallowing index because some of our patients were unable to understand and complete the EAT-10 questionnaire. Due to its complexity and being time-consuming, our study introduced a simplified questionnaire for evaluating swallowing ability in older adults. The questionnaire comprises only 4-item questions with a dichotomous answer, which is simpler than the 10-item questions answered using the 5-point Likert scale in the EAT-10.

Dependent status was significantly associated with low swallowing ability and malnutrition risk. Although the dependency level was associated with advanced age, a higher age was associated with lower tongue strength, but not having a swallowing problem. As supported by previous studies in healthy adults and older people, maximum tongue strength reduced with

advanced age [17-19], which might be due to reduced musculoskeletal function[20] and masticatory muscle strength [17]. These findings imply that dependency status has a greater influence on swallowing ability than chronological age. Therefore, maintaining functional health and being active are important to prevent the progression of oral hypofunction in older adults.

The number of remaining teeth, posterior occlusal support, and denture type were not associated with the subjective or objective swallowing indices. Previous studies found that maximum tongue pressure increased with greater posterior occlusal support assessed using the Eicher index [17, 18]. In the present study, however, all edentulous patients wore a dental prosthesis when performing the tongue strength measurement because most of them required anterior denture teeth to position the pressure bulb. Wearing a dental prosthesis increases the number of posterior occlusal contacts, and therefore, enhances the bite force in edentulous individuals [21]. Individuals with higher occlusal forces present higher masticatory muscle strength [17], which is associated with lower dysphagia risk [12, 22]. Thus, wearing a dental prosthesis might reduce malnutrition risk in edentulous older adults regardless of the remaining functional teeth and posterior occlusal support.

In accordance with previous studies in middle-aged and older adults, malnutrition risk was associated with low tongue strength [3, 23]. To categorize low and high tongue strength, our study chose a cut-off value of 18 kPa because it gave the highest AUC value when plotting the ROC curve. The Japanese Society of Gerodontology suggests using 30 kPa as a cut-off value to diagnose decreased tongue strength [2]. Furthermore, a study in Canadian older adults in long-term care used a value of 26 kPa, the average tongue pressure of the study samples, as the cut-off value to categorize tongue pressure into low and high levels [3]. In our study, however, using either 30- or 26-kPa tongue pressure as a cut-off value gave relatively low sensitivity and PPV in

estimating malnutrition risk. Because the thickness of swallowing muscles might be different among ethnicities [24], individual studies may need to identify the ethnic-specific normal values of tongue strength.

The sensitivity of the subjective and objective swallowing indices in estimating malnutrition risk was comparable. However, the PPV value of the subjective index was about 1.8-fold greater than that of tongue strength. Moreover, the AUC obtained from the subjective swallowing index and malnutrition risk was 15.2% higher than the objective tongue strength value. These findings indicated that the subjective swallowing index might be a more appropriate tool for estimating malnutrition risk in older adults. As supported by earlier studies [11, 25], recognizing signs and symptoms with a thorough history taking is key in early diagnosis and detecting swallowing impairment. Tongue strength measurement could be a supplemental tool to confirm the subjective finding whenever patients or their caregivers have communication problems or are unaware of the symptoms.

Treating oral frailty and oral hypofunction requires a multidisciplinary approach. Thus, dental professionals can be part of a holistic team by early detection of declined swallowing function to prevent the progression into the irreversible dysfunction stage. This study suggests using a simplified 4-item questionnaire as a screening method for evaluating swallowing ability in older adults that do not require an experienced physician in routine dental practice. In addition, we propose a concept for identifying a cut-off value to categorize lower and higher tongue strength using malnutrition risk as an outcome. However, further study in a larger population is required to verify the reliability and validity of the questionnaire. Further use of the simplified questionnaire for early detection of swallowing problem in a clinic and community-based study by caregiver and non-healthcare personnel should be evaluated.

Conclusions

The 4-item self-reported swallowing problems questionnaire was developed as a subjective index for evaluating swallowing ability in older adults. The convergent validity of the subjective index was verified using objective tongue strength as a reference. Both subjective and objective indices were associated with malnutrition risk in older adults. However, the subjective swallowing index better estimated malnutrition risk than the objective tongue strength.

Acknowledgment

The authors gratefully acknowledge Dr. Kevin Tompkins for language revision of the manuscript.

Funding

This research is funded by Chulalongkorn University, Grant number CU_GR_63_11_32_04. The funders had no role in study design, data collection and analysis, decision to publish, or preparation of the manuscript

Conflict of interest

The authors declare no potential conflict of interest and no competing interest in this study

Data availability statement

All relevant data are within the paper and its supporting information files.

References

1. Razak PA, Richard KM, Thankachan RP, Hafiz KA, Kumar KN, Sameer KM. Geriatric oral health: a review article. J Int Oral Health 2014;6(6):110-116.
2. Minakuchi S, Tsuga K, Ikebe K, Ueda T, Tamura F, Nagao K, et al. Oral hypofunction in the older population: Position paper of the Japanese Society of Gerodontology in 2016. Gerodontology 2018;35(4):317-324.
3. Namasivayam-MacDonald AM, Morrison JM, Steele CM, Keller H. How Swallow Pressures and Dysphagia Affect Malnutrition and Mealtime Outcomes in Long-Term Care. Dysphagia 2017;32(6):785-796.
4. Sura L, Madhavan A, Carnaby G, Crary MA. Dysphagia in the elderly: management and nutritional considerations. Clin Interv Aging 2012;7(287-298.
5. Rasheed S, Woods RT. Malnutrition and quality of life in older people: a systematic review and meta-analysis. Ageing Res Rev 2013;12(2):561-566.
6. Clark HM, Henson PA, Barber WD, Stierwalt JA, Sherrill M. Relationships among subjective and objective measures of tongue strength and oral phase swallowing impairments. Am J Speech Lang Pathol 2003;12(1):40-50.
7. Chatindiara I, Allen J, Popman A, Patel D, Richter M, Kruger M, Wham C. Dysphagia risk, low muscle strength and poor cognition predict malnutrition risk in older adults athospital admission. BMC Geriatr 2018;18(1):78.
8. Hansen T, Kjaersgaard A. Item analysis of the Eating Assessment Tool (EAT-10) by the Rasch model: a secondary analysis of cross-sectional survey data obtained among community-dwelling elders. Health Qual Life Outcomes 2020;18(1):139.

9. Wilmskoetter J, Bonilha H, Hong I, Hazelwood RJ, Martin-Harris B, Velozo C. Construct validity of the Eating Assessment Tool (EAT-10). Disabil Rehabil 2019;41(5):549-559.

10. Guigoz Y. The Mini Nutritional Assessment (MNA) review of the literature--What does it tell us? J Nutr Health Aging 2006;10(6):466-485; discussion 485-467.

11. Nawaz S, Tulunay-Ugur OE. Dysphagia in the Older Patient. Otolaryngol Clin North Am 2018;51(4):769-777.

12. Hara K, Tohara H, Wada S, Iida T, Ueda K, Ansai T. Jaw-opening force test to screen for Dysphagia: preliminary results. Arch Phys Med Rehabil 2014;95(5):867-874.

13. Machida N, Tohara H, Hara K, Kumakura A, Wakasugi Y, Nakane A, Minakuchi S. Effects of aging and sarcopenia on tongue pressure and jaw-opening force. Geriatr Gerontol Int 2017;17(2):295-301.

14. Hayashi R, Tsuga K, Hosokawa R, Yoshida M, Sato Y, Akagawa Y. A novel handy probe for tongue pressure measurement. Int J Prosthodont 2002;15(4):385-388.

15. Rockwood K, Song X, MacKnight C, Bergman H, Hogan DB, McDowell I, Mitnitski A. A global clinical measure of fitness and frailty in elderly people. CMAJ 2005;173(5):489-495.

16. Thai Cognitive Test Development Committee 1999. Mini-Mental State Examination-Thai 2002 Bangkok: Institute of Geriatric Medicine, Department of Medical Services, Ministry of Public Health, Thailand; 2002.

17. Hara K, Tohara H, Kenichiro K, Yamaguchi K, Ariya C, Yoshimi K, Nakane A, Minakuchi S. Association between tongue muscle strength and masticatory muscle strength. J Oral Rehabil 2019;46(2):134-139.

18. Kikutani T, Tamura F, Nishiwaki K, Kodama M, Suda M, Fukui T, et al. Oral motor function and masticatory performance in the community-dwelling elderly. Odontology 2009;97(1):38-42.

19. Higa C, Mori T, Hiraoka A, Takeda C, Kuroki A, Yoshikawa M, Yoshida M, Tsuga K. Five-year change in maximum tongue pressure and physical function in community-dwelling elderly adults. J Dent Sci 2020;15(3):265-269.

20. Kletzien H, Cullins MJ, Connor NP. Age-related alterations in swallowing biomechanics. Exp Gerontol 2019;118(45-50.

21. Limpuangthip N, Somkotra T, Arksornnukit M. Subjective and objective measures for evaluating masticatory ability and associating factors of complete denture wearers: A clinical study. J Prosthet Dent 2021;125(2):287-293.

22. Maeda K, Akagi J. Decreased tongue pressure is associated with sarcopenia and sarcopenic dysphagia in the elderly. Dysphagia 2015;30(1):80-87.

23. Nakazawa Y, Kikutani T, Igarashi K, Yajima Y, Tamura F. Associations between tongue strength and skeletal muscle mass under dysphagia rehabilitation for geriatric out patients. J Prosthodont Res 2020;64(2):188-192.

24. Jensen B, Moritoyo T, Kaufer-Horwitz M, Peine S, Norman K, Maisch MJ, et al. Ethnic differences in fat and muscle mass and their implication for interpretation of bioelectrical impedance vector analysis. Appl Physiol Nutr Metab 2019;44(6):619-626.

25. Malagelada J, Bazzoli F, Boeckxstaens G, De Looze D, Fried M, Kahrilas P, et al. Dysphagia: Global Guidelines and Cascades. World Gastroenterology Organisation 2014.

Click here to access/download
Supporting Information
Swallow_PlosOne.xlsx
