# Supplementary material for: A simplified method for evaluating swallowing ability and estimating malnutrition risk: A pilot study in older adults
Source: PLoS One. 2022 Feb 16;17(2):e0263896. doi: 10.1371/journal.pone.0263896 (PMC8849596; doi:10.1371/journal.pone.0263896)
Supplement: Attachment — Submitted filename: PLOS ONE Second Revision.pdf [file pone.0263896.s007.pdf]

PLOS ONE

A simplified method for evaluating swallowing ability and estimating malnutrition risk: A pilot study in older adults

--Manuscript Draft--

**Manuscript Number:**	PONE-D-21-21718R1
**Article Type:**	Research Article
**Full Title:**	A simplified method for evaluating swallowing ability and estimating malnutrition risk: A pilot study in older adults
**Short Title:**	Swallowing ability and malnutrition risk
**Corresponding Author:**	Orapin Komin, Ph.D.<br>Chulalongkorn University Faculty Of Dentistry<br>Bangkok, Thailand THAILAND
**Keywords:**	Nutritional assessment; oral function; Swallowing function; Tongue function; Tongue pressure.
**Abstract:**	Objectives: The aim of this pilot study was to develop a Thai-version of a simple swallowing questionnaire, called the T-SSQ, and to evaluate the association between malnutrition risk and swallowing ability, determined objectively by tongue strength and subjectively by the T-SSQ. Sensitivity analysis was also performed to determine which swallowing indices better estimate malnutrition in older adults.<br>Methods: This cross-sectional study comprised two phases: Phase I, development and cross-cultural translation of the T-SSQ; and Phase II, application of the T-SSQ in 60 older adults. In Phase I, content and face validity of the T-SSQ was evaluated by 10 experts and 15 older adults. In Phase II, the convergent validity of the T-SSQ was evaluated by determining its association with objective tongue strength. Nutritional status was evaluated using the Thai-version of the Mini-Nutritional Assessment. Covariates included sociodemographic characteristics, and oral and health-related status. Adjusting for covariates, the associations between the two swallowing indices and malnutrition risk were determined using multivariable regression analyses. A cut-off value for low tongue strength was determined using a receiver operating characteristic (ROC) curve, and sensitivity analysis between the swallowing indices and malnutrition risk was performed.<br>Results: The T-SSQ comprised 4-items of common signs and symptoms of a swallowing problem. Its content and face validity were verified. Older adults were considered as having a swallowing problem when at least one item was reported. Convergent validity of the subjective index was shown by significantly different tongue strength values between the participants with and without a swallowing problem (p for independent t-test = 0.014). Based on the highest area under the ROC curve, an 18-kPa cut-off value was chosen to classify low tongue strength. Having a swallowing problem and low tongue strength was significantly associated with malnutrition risk. The positive predictive value of the subjective swallowing index was 1.8-fold higher than objective tongue strength.<br>Conclusions: Self-reported swallowing problems determined by the T-SSQ can be used as a subjective index for evaluating swallowing ability in older adults. Subjective swallowing problems and objective tongue strength were associated with malnutrition risk. However, the T-SSQ estimated malnutrition risk better than the objective index.
**Order of Authors:**	Nareudee Limpuangthip
	Orapin Komin, Ph.D.
	Teerawut Tatiyapongpaiboon
**Response to Reviewers:**	Response to reviewers<br><br>We are pleased to submit our revised manuscript Number: PONE-D-21-21718. The title has been modified to ‘A simplified method for evaluating swallowing ability and estimating malnutrition risk: A pilot study in older adults.’ The newly developed questionnaire has been named ‘Thai-version of Simplified Swallowing Questionnaire’ or ‘T-SSQ’. The requested revisions have been made in the manuscript in track

Powered by Editorial Manager® and ProduXion Manager® from Aries Systems Corporation

changes, and our point-by-point responses are below.

Reviewer No. 1:
Reviewer point #1: The authors have embarked on a novel method to identify a screening tool for evaluating swallowing ability in older adults. The manuscript is well written and systematically presented.
However, the conclusions made from this study needs further justification for application on a larger population because its reliability and validity is yet unclear. Probably, this can be introduced as a pilot study to further analyze on the sensitivity of the proposed 4 items self-reported assessment tool.
Author response #1: Thank you for your comment. We have revised our study limitations, further study suggestions and conclusion. Due to a limitation, the study has been changed into a pilot study.
Reviewer point #2: Things that need further clarification include the selection of the items, independence of each item from the other, the scoring value adopted for this tool, the measurement equivalence of the tool, etc.
Author response #2: Development of the T-SSQ including the selection of the items, the scoring value adopted for the T-SSQ, its interpretation, and its validity testing have been clarified in the Materials and Methods section (Phase I subsection, Page 5-6). We did not use any statistical test to evaluate the independence of each item from the other because the items were selected by experts.
Reviewer point #3: The authors do not mention in which language the questionnaire was administered; if in the local language, any translation was done and cross-culturally validated.
Author response #3: Thai language was used for the swallowing questionnaire. The cross-cultural translation from English to Thai version was performed, and the descriptions have been added in the Materials and Methods section (Phase I subsection, Page 5)
Reviewer point #4: The cut off value for the Objective Swallowing Assessment was way below that reported in cited literature and in case that cut-off is raised there is a statistically significant variation between the Objective and Subjective Swallowing tools applied in this study.
Author response #4: The descriptions about the cut-off value have been revised in the Discussion section (Page 15-16) according to the reviewer's suggestion.
Reviewer point #5: The sample size in this study needs to be evaluated if is adequate to introduce a sensitive assessment tool that can be used as a key instrument in early diagnosis and detecting of swallowing impairment among older adults.
Author response #5: The power analysis of the sample size has been revised by evaluating whether the T-SSQ was a sensitive assessment tool for using as a key instrument in early diagnosis and detecting swallowing impairment among older adults. The revisions have been made in the Materials and Methods section (Power analysis subsection, Page 9)

Reviewer No. 2:
Reviewer point #1: Control group should have been included.
Author response #1: Our pilot study did not include patients who were diagnosed with dysphagia by physicians. Therefore, a positive control group was not present in this study. This was because we wanted to develop a screening tool for swallowing ability impairment rather than a tool for dysphagia diagnosis. This limitation has been added in the Discussion section (Page 17).
Reviewer point #2: Newly developed questionnaire should be validated. Statistics data should be furnished completely.
Author response #2: Descriptions about the validation of the newly developed questionnaire (T-SSQ) has been added in the Materials and Methods section (Phase I subsection). The statistical analysis has also been revised.
Reviewer point #3: Relevant figures with legends to be provided. Copy of questionnaire should be given.
Author response #3: The relevant figures with legends have been provided in the manuscript file. A copy of the questionnaire (in Thai, and English translation) has been provided as a supplementary file.

Additional responses to the comments in the attached Pdf files:
Materials and Methods section
-The references of the Thai-version of the Mini-Nutritional Assessment (MNA) has

	been revised.<br>-The references for the tongue pressure measurement and the Thai-version of Mini-Mental State Evaluation (MMSE) have been added.<br>-To evaluate the reliability of the T-SSQ in older adults, the inter-examiner reliability was examined in 15 patients at the patients' first evaluation visit. Test-retest reliability was evaluated by reinterviewing these patients one week later. The descriptions have been revised in the Swallowing ability assessment subsection (Page 8).<br>Results section<br>-The results for the descriptive statistics have been demonstrated in the Result section and Tables.<br>-The statistical tests have been added as a footnote of Table 1.<br>Discussion section<br>-The patients who were unable to perform a tongue pressure test due to severely declined functional or intellectual conditions were excluded. This exclusion criterion has been included in the Materials and Methods section (Page 5).<br>-The study limitations have been added according to the reviewers' suggestions.<br><br><br>Sincerely yours<br>Orapin Komin<br>Corresponding author
**Additional Information:**	
**Question**	**Response**
**Financial Disclosure**<br><br>Enter a financial disclosure statement that describes the sources of funding for the work included in this submission. Review the submission guidelines for detailed requirements. View published research articles from *PLOS ONE* for specific examples.<br><br>This statement is required for submission and **will appear in the published article** if the submission is accepted. Please make sure it is accurate.	Yes. This research is funded by Chulalongkorn University, Grant number CU_GR_63_11_32_04. The funders had no role in study design, data collection and analysis, decision to publish, or preparation of the manuscript.

**Unfunded studies**<br>Enter: *The author(s) received no specific funding for this work.*<br><br>**Funded studies**<br>Enter a statement with the following details:<br>• Initials of the authors who received each award<br>• Grant numbers awarded to each author<br>• The full name of each funder<br>• URL of each funder website<br>• Did the sponsors or funders play any role in the study design, data collection and analysis, decision to publish, or preparation of the manuscript?<br>• **NO** - Include this sentence at the end of your statement: *The funders had no role in study design, data collection and analysis, decision to publish, or preparation of the manuscript.*<br>• **YES** - Specify the role(s) played.<br><br>* typeset	
**Competing Interests**<br><br>Use the instructions below to enter a competing interest statement for this submission. On behalf of all authors, disclose any competing interests that could be perceived to bias this work—acknowledging all financial support and any other relevant financial or non-financial competing interests.<br><br>This statement is required for submission and **will appear in the published article** if the submission is accepted. Please make sure it is accurate and that any funding sources listed in your Funding Information later in the submission form are also declared in your Financial Disclosure statement.<br><br>View published research articles from *PLOS ONE* for specific examples.	The authors have declared that no competing interests exist.

**NO authors have competing interests**<br><br>Enter: *The authors have declared that no competing interests exist.*<br><br>**Authors with competing interests**<br><br>Enter competing interest details beginning with this statement:<br><br>*I have read the journal's policy and the authors of this manuscript have the following competing interests: [insert competing interests here]*<br><br>* typeset	
**Ethics Statement**<br><br>Enter an ethics statement for this submission. This statement is required if the study involved:<br><br>• Human participants<br>• Human specimens or tissue<br>• Vertebrate animals or cephalopods<br>• Vertebrate embryos or tissues<br>• Field research<br><br>Write "N/A" if the submission does not require an ethics statement.<br><br>General guidance is provided below. Consult the submission guidelines for detailed instructions. **Make sure that all information entered here is included in the Methods section of the manuscript.**	The study protocol was approved by the Ethics Committee of the Faculty of Dentistry, Chulalongkorn University (HREC-DCU 2018-112).

Powered by Editorial Manager® and ProduXion Manager® from Aries Systems Corporation

**Format for specific study types**<br><br>**Human Subject Research (involving human participants and/or tissue)**<br>• Give the name of the institutional review board or ethics committee that approved the study<br>• Include the approval number and/or a statement indicating approval of this research<br>• Indicate the form of consent obtained (written/oral) or the reason that consent was not obtained (e.g. the data were analyzed anonymously)<br><br>**Animal Research (involving vertebrate animals, embryos or tissues)**<br>• Provide the name of the Institutional Animal Care and Use Committee (IACUC) or other relevant ethics board that reviewed the study protocol, and indicate whether they approved this research or granted a formal waiver of ethical approval<br>• Include an approval number if one was obtained<br>• If the study involved *non-human primates*, add *additional details* about animal welfare and steps taken to ameliorate suffering<br>• If anesthesia, euthanasia, or any kind of animal sacrifice is part of the study, include briefly which substances and/or methods were applied<br><br>**Field Research**<br><br>Include the following details if this study involves the collection of plant, animal, or other materials from a natural setting:<br>• Field permit number<br>• Name of the institution or relevant body that granted permission	
**Data Availability**<br><br>Authors are required to make all data underlying the findings described fully available, without restriction, and from the time of publication. PLOS allows rare exceptions to address legal and ethical concerns. See the PLOS Data Policy and FAQ for detailed information.	No - some restrictions will apply

A Data Availability Statement describing where the data can be found is required at submission. Your answers to this question constitute the Data Availability Statement and **will be published in the article**, if accepted.<br><br>**Important:** Stating 'data available on request from the author' is not sufficient. If your data are only available upon request, select 'No' for the first question and explain your exceptional situation in the text box.<br><br>Do the authors confirm that all data underlying the findings described in their manuscript are fully available without restriction?	
**Describe where the data may be found in full sentences. If you are copying our sample text, replace any instances of XXX with the appropriate details.**<br><br>• If the data are **held or will be held in a public repository**, include URLs, accession numbers or DOIs. If this information will only be available after acceptance, indicate this by ticking the box below. For example: *All XXX files are available from the XXX database (accession number(s) XXX, XXX.*).<br>• If the data are all contained **within the manuscript and/or Supporting Information files**, enter the following: *All relevant data are within the manuscript and its Supporting Information files.*<br>• If neither of these applies but you are able to provide **details of access elsewhere**, with or without limitations, please do so. For example:<br><br>*Data cannot be shared publicly because of [XXX]. Data are available from the XXX Institutional Data Access / Ethics Committee (contact via XXX) for researchers who meet the criteria for access to confidential data.*<br><br>*The data underlying the results presented in the study are available from (include the name of the third party*	All relevant data are within the manuscript and its Supporting Information files.

and contact information or URL).<br>• This text is appropriate if the data are owned by a third party and authors do not have permission to share the data.<br><br>* typeset	
Additional data availability information:	Tick here if the URLs/accession numbers/DOIs will be available only after acceptance of the manuscript for publication so that we can ensure their inclusion before publication.

Powered by Editorial Manager® and ProduXion Manager® from Aries Systems Corporation

Manuscript Click here to access/download;Manuscript;Manuscript.docx

Title page

A simplified method for evaluating swallowing ability and estimating malnutrition risk: A pilot study in older adults

Short title: Swallowing ability and malnutrition risk

Nareudee Limpuangthip[1¶], Orapin Komin[1¶*], Teerawut Tatiyapongpaiboon[2]

[1]Department of Prosthodontics, Faculty of Dentistry, Chulalongkorn University, Bangkok, Thailand.

[2]Thungyai Hospital, Nakhon Si Thammarat, Thailand

[¶]These authors equally contributed to this study

*** Corresponding author:**

E-mail: Orapin.geriatric@gmail.com

Abstract

Objectives: The aim of this pilot study was to develop a Thai-version of a simple swallowing questionnaire, called the T-SSQ, and to evaluate the association between malnutrition risk and swallowing ability, determined objectively by tongue strength and subjectively by the T-SSQ. Sensitivity analysis was also performed to determine which swallowing indices better estimate malnutrition in older adults.

Methods: This cross-sectional study comprised two phases: Phase I, development and cross-cultural translation of the T-SSQ; and Phase II, application of the T-SSQ in 60 older adults. In Phase I, content and face validity of the T-SSQ was evaluated by 10 experts and 15 older adults. In Phase II, the convergent validity of the T-SSQ was evaluated by determining its association with objective tongue strength. Nutritional status was evaluated using the Thai-version of the Mini-Nutritional Assessment. Covariates included sociodemographic characteristics, and oral and health-related status. Adjusting for covariates, the associations between the two swallowing indices and malnutrition risk were determined using multivariable regression analyses. A cut-off value for low tongue strength was determined using a receiver operating characteristic (ROC) curve, and sensitivity analysis between the swallowing indices and malnutrition risk was performed.

Results: The T-SSQ comprised 4-items of common signs and symptoms of a swallowing problem. Its content and face validity were verified. Older adults were considered as having a swallowing problem when at least one item was reported. Convergent validity of the subjective index was shown by significantly different tongue strength values between the participants with and without a swallowing problem (p for independent t-test = 0.014). Based on the highest area

under the ROC curve, an 18-kPa cut-off value was chosen to classify low tongue strength. Having a swallowing problem and low tongue strength was significantly associated with malnutrition risk. The positive predictive value of the subjective swallowing index was 1.8-fold higher than objective tongue strength.

Conclusions: Self-reported swallowing problems determined by the T-SSQ can be used as a subjective index for evaluating swallowing ability in older adults. Subjective swallowing problems and objective tongue strength were associated with malnutrition risk. However, the T-SSQ estimated malnutrition risk better than the objective index.

Keywords: Nutritional assessment, Oral function, Swallowing function, Tongue function, Tongue pressure.

Introduction

Oral and general health functionally decline as people age [1]. Gradually declined oral function can lead to oral frailty followed by oral hypofunction. However, they can recover to the healthy stage by early detection and proper dental treatment [2]. Oral health becomes oral frailty when a person has decreased occluding teeth, increased unchewable foods, or slight choking/spillage while eating. Moreover, oral hypofunction is diagnosed when 3 out of 7 oral signs or symptoms are present: oral uncleanness and dryness, reduced occlusal force, reduced chewing function, reduced tongue and lip motor function, and reduced tongue pressure and swallowing function [2]. Because eating and swallowing ability plays a major role in oral function, a decline in swallowing ability contributes to malnutrition [3, 4]. Malnutrition increases the risk of morbidity and mortality, and negatively affect the quality of life of older adults [5].

To prevent malnutrition in older adults, early detection of declined swallowing ability is necessary. Several objective and subjective indices have been used to evaluate swallowing ability in older adults. Tongue pressure measurement is commonly used to objectively evaluate swallowing ability, because tongue motor function plays an important role in mastication and swallowing [3, 6]. However, this method requires special instruments and time to perform. To evaluate swallowing ability subjectively, the 10-item Eating Assessment Tool (EAT-10) is commonly used because it is considered a reliable and validated questionnaire [2, 6, 7]. However, some studies reported the limitations of EAT-10 regarding its substantial floor effect, several redundant items, and relatively low construct validity [8, 9]. Thus, an alternative simple screening method for evaluating swallowing ability should be proposed for the early detection of oral function when a patient is in the frailty or hypofunction stage.

The aims of the present study were to develop a Thai-version of a simplified swallowing questionnaire (T-SSQ), and to evaluate the association between malnutrition risk and swallowing ability, determined objectively by tongue strength and subjectively by the T-SSQ. In addition, sensitivity analysis was performed to determine which swallowing indices better estimate malnutrition in older adults.

Materials and Methods

Study design and participants

The present study was a cross-sectional design. The study protocol was approved by the Ethics Committee of the Faculty of Dentistry, Chulalongkorn University (HREC-DCU 2018-

112). The participants and their guardians provided written informed consent prior to participating in the study.

The participants were older adults (aged ≥ 60 years old) recruited from patients who received dental treatment at the Geriatric and Special Patients Care Clinic, Faculty of Dentistry, Chulalongkorn University during August 2017– January 2019. The exclusion criteria were patients who declined or were unable to perform a tongue pressure test due to severely declined functional or intellectual conditions, or currently had malnutrition.

Phase I. Development of simplified swallowing questionnaire

A 9-item swallowing questionnaire (SQ) was created based on the common signs and symptoms of dysphagia patients according to Walker et al (1990) [10] and Nawaz S & Tulunay-Ugur OE (2018) [11] (Supplementary file 1). Cross-cultural translation of the SQ was performed according to the WHO guidelines [12]. A forward translation from English to the Thai version was conducted by two-independent translators, one dentist and one non-dentist, and integrated into a single Thai version. A back translation from Thai to English version was then conducted by two-independent translators, one was a dentist and the other was not. To verity the content validity of the translated version, the principal investigators and translators discussed any discrepancies related to the meaning of words and phrases between the Thai and English versions. The investigators ensured that the basic concepts and meanings of all terms with reference to the original version were maintained, and then, the Thai-version of the swallowing questionnaire (T-SQ) was proposed.

The content validity of the T-SQ was evaluated by 10 experts (5 physicians and 5 dentists). They gave responses whether they agreed that each of the 9 items indicated a swallowing problem (agree, disagree). The items with less than 80% agreement were excluded, and the final version was reduced to 4 items. A face validity of the 4-item swallowing questionnaire was evaluated in 20 older adults (age range 52–77 years). They gave responses whether they clearly understood the description of each item using a dichotomous scale (clearly understood and unclear), and provided suggestions if any rephrasing was necessary for easier understanding. All items received 90–100% clearly understood response but with some suggestions. After the discrepancies were discussed, a final version of the Thai-version simplified swallowing questionnaire (T-SSQ) was developed. The word liquid in the English version was translated into naam in the Thai version, which means water (noun) or related to liquid substance (adjective) for better understanding by Thais.

The T-SSQ comprised 4 items: 1) having problems swallowing certain food or liquids, or could not swallow at all, 2) coughing or choking when eating or drinking, 3) aspirations with liquids or solid food occurs, and 4) a sensation that food got stuck in the throat or chest. The response to each item was given as having (1) or not having (0) a problem. A person was defined as having a swallowing problem when at least one of items was present at least once a week during the past month.

Phase II. Application of the T-SSQ

Phase II, the T-SSQ application, was conducted as a pilot study in 60 older adults who did not participate in Phase I. Swallowing ability was assessed subjectively by the T-SSQ and objectively by tongue strength. Convergent validity of the T-SSQ was evaluated using objective

tongue pressure as a reference. Sensitivity analysis was performed to determine which subjective and objective swallowing indices better estimate malnutrition in older adults.

Nutritional assessment

Nutritional status was measured using the Thai-version of the Mini-Nutritional Assessment (MNA) with a score ranging from 0–30. The participants were categorized as having malnutrition risk when MNA score = 17–23.5, and being normal when the score ≥24. The MNA was used because it is a standardized and validated instrument in older adults [13, 14].

Swallowing ability assessment

Subjective swallowing ability was evaluated using the T-SSQ developed in Phase I. The participants were interviewed with the assistance of their caregivers, if present. The participants were defined as having a swallowing problem when at least one of the T-SSQ items was reported. The inter-examiner reliability was determined using 15 patients at the patients' first evaluation visit. The test-retest reliability was evaluated by reinterviewing the 15 patients one week later. The weighted Kappa scores for the inter-examiner and test-retest reliability assessments were approximately 0.82 and 0.87, respectively.

The objective swallowing ability was evaluated through tongue pressure, measured using the JMS TPM-02 measurement device (JMS, Inc., Hiroshima, Japan), which consisted of a plastic catheter and a balloon. The participants sat in an upright position. The balloon was inserted into their oral cavity and placed on the anterior part of the palate with their lips and jaw closed, while the plastic catheter was held at the midpoint of the central incisors. The participants raised their tongue and pressed the balloon against the hard palate as hard as possible, and the maximum tongue pressure (kPa, kilopascal) was read. This procedure was done in triplicate with

5 min resting intervals, and the tongue strength (kPa) was calculated from the average value of the three measurements [15-17].

Covariates

Information regarding biological factors, oral- and health-related status was recorded. Biological factors were an individuals' age and sex. A dentist evaluated oral status, comprising the number of remaining functional teeth (ranged from 0 to 28 teeth), number of posterior occluding pairs (ranged 0–8 pairs), and type of denture worn. If more than one type of denture was present, it was classified as the type with the higher number of tooth loss.

Health-related status covered the participants physical and psychological conditions: dependency status and cognitive status, respectively. The clinical frailty scale (CFS) was used to categorize dependency status into independent, semi-dependent, and dependent [18]. Cognitive function was evaluated using the Thai-version of the Mini-Mental State Evaluation (MMSE). With a score ranging from 0–30, the participants were considered as having mild cognitive impairment (MCI) when the score was below 18 and 22 when their educations was at least primary and above primary level, respectively [19].

Power analysis

A power analysis of the sample size was performed to determine whether the T-SSQ was a sensitive assessment tool for using as a key instrument in early diagnosis and detecting swallowing impairment among older adults. The results indicated that the tongue strength (mean ±s.d.) of the participants who reported a swallowing problem (n_1=7) and those who did not (n_2=54) were 16.8 ±10.6 kPa and 26.7 ±9.6 kPa, respectively. Using the two-independent means test, a power of 70% was calculated at 5% type I error.

Data analysis

Descriptive statistics was performed to determine the percentage (%) and mean ±standard deviation (s.d.). Univariate analyses of the associations between related variables and having a swallowing problem were analyzed using the chi-squared test, whereas its association with tongue strength and MNA score were analyzed using either one-way ANOVA or independent t-test. Variables with p-value < 0.10 were included in the multivariable analyses. Adjusting for covariates, multivariable logistic and linear regression were used to determine the factors associated with the subjective and objective swallowing indices, and their associations with malnutrition risk. To determine the convergent validity, the tongue strength values of the participants with and without swallowing problem assessed by the T-SSQ were compared using the independent t-test. A receiver operating characteristic (ROC) curve was plotted to determine the area under the curve (AUC) in the malnutrition risk models; the higher the AUC, the better the model was able to distinguish between the participants with and without malnutrition risk. To categorize the low and high tongue strength, a cut-off value that gave the highest AUC value was chosen. For the sensitivity analysis, the positive predictive value (PPV), negative predictive value (NPV), sensitivity, and specificity between malnutrition risk and the two swallowing ability indices were calculated. The data were analyzed using STATA version 13.0 (StataCorp LP) at a 5% significance level.

Results

The T-SSQ was developed as a subjective swallowing index, comprising 4-items of common signs and symptoms of a swallowing problem. The content and face validity of the T-

SSQ was assessed. The characteristics of the participants attending in Phase II based on the subjective and objective swallowing indices, and malnutrition risk are shown in Table 1. Their mean ±s.d. age was 78.0 ±7.0 years old. Malnutrition risk was found in 18% of the participants, while the others were within normal limits. MCI was present in 90% of the semi- and dependent older participants. Oral status was associated with the subjective and objective swallowing indices, and malnutrition risk. The convergent validity of the T-SSQ was revealed as shown by a significant difference in tongue strength values between the participants with and without a swallowing problem (p for independent t-test = 0.014).

Table 1. Characteristics of the study participants.

Variables	Overall distribution: %	Swallowing ability index: Self-reported swallowing problem by T-SSQ (Yes): %	Swallowing ability index: Maximum tongue pressure (kPa): mean (±s.d.)	Nutritional status: MNA score: mean (±s.d.)
Overall	***100.0***	***11.7***	***25.5 (±10.1)***	***26.0 (±3.0)***
Age (years): 60 – 69	15.0	11.1	34.5 (±8.7)	25.0 (±3.8)
70 – 79	40.0	4.2	26.8 (±9.3)*	26.5 (±2.1)
80 and above	45.0	18.5†	21.4 (±9.4)*	26.0 (±3.3)
Sex: Male	48.3	10.3	27.7 (±9.8)	26.9 (±2.1)
Female	51.7	12.9	23.5 (±10.1)†	25.2 (±3.4)
**Health-related status**				
Dependency status: Independent	71.7	4.6	27.8 (±9.8)	26.9 (±2.1)
Semi-dependent	18.3	27.3*	19.1 (±8.9)*	25.1 (±2.5)
Dependent	10.0	33.3*	21.2 (±8.8)*	21.1 (±3.9)*
MMSE score: Normal	78.3	6.2	26.5 (±10.4)	26.5 (±2.2)
Mild cognitive impairment	21.7	30.8*	22.1 (±8.6)†	24.1 (±4.4)
**Oral status**				
Natural teeth: 0	19.7	16.7	24.1 (±10.2)	26.0 (±3.6)
1–19	70.5	11.6	25.2 (±9.7)	25.9 (±3.0)
20 and above	9.8	0.0	30.1 (±9.9)	27.0 (±2.0)
Posterior occluding pairs: 0	76.7	13.0	24.5 (±10.2)	26.1 (±3.0)
1–3	13.3	12.5	32.4 (±10.7)	25.9 (±1.8)
4 and above	10.0	0.0	26.7 (±9.6)	25.4 (±4.5)
Denture type: No denture	3.3	0.0	30.7 (±1.8)	29.2 (±1.1)
Removable partial denture	45.0	7.4	27.3 (±8.8)	26.0 (±2.7)
Complete denture	51.7	16.1	23.6 (±11.1)	25.8 (±3.2)
**Swallowing ability**				
Having swallowing problem: No	11.7	-	26.7 (±9.6)	26.0 (±2.8)
Yes	88.3		16.8 (±10.6)*	23.0 (±2.7)*

*p <0.05, †p<0.10 determined by independent t-test or one-way ANOVA and post-hoc comparison test.

.

There were significant associations between the subjective and objective swallowing indices and malnutrition risk after adjusting for potential covariates (Table 2). Because there was collinearity between dependency status and MCI, the MCI variable was not included in the multivariable regression models. Based on the ROC curve, 18 kPa was chosen as a cut-off value to categorize the participants into low and high tongue strength because it gave the highest AUC value when plotting the curve between tongue strength and malnutrition risk (Figure 1).

Table 2. The associations between swallowing indices and related variables.

Variables	Swallowing ability index		Malnutrition risk: adjusted OR (95% CI)	
	**Self-reported swallowing problem by T-SSQ (Yes): adjusted OR (95% CI)**	**Maximum tongue pressure (kPa): adjusted β (95% CI)**	**Model 1**	**Model 2**
Age (years old)	1.05 (0.92, 1.18)	-0.65 (-1.00, -0.30)*	1 (ref)	0.98 (0.84, 1.14)
Sex (Female)	0.55 (0.07, 4.38)	-1.00 (-5.76, 3.76)	6.28 (0.44, 45.4)	3.23 (0.45, 23.4)
Dependency status: Independent	1 (ref)	1 (ref)	1 (ref)	1 (ref)
Semi-dependent	8.44 (0.89.30.1)†	-4.19 (-10.6, 2.28)	0.21 (0.01, 6.26)	0.80 (0.07, 8.65)
Dependent	13.4 (1.24, 39.4)*	-6.67 (-14.1, 1.18)†	22.3 (1.53, 53.3)*	40.6 (3.30, 85.3)*
**Swallowing ability measures**				
Self-reported swallowing problem (Yes)			35.5 (3.49, 75.5)*	-
Maximum tongue pressure (<18 kPa)			-	0.11 (0.01, 0.71)*
AUC (%)			***88.3***	***74.9***

*p <0.05, †p<0.10. OR, odds ratio; β, beta-coefficient; CI, confidence interval; ref, reference; AUC, Area under the Receiver Operating Characteristic (ROC) curve.

Figure 1. Receiver Operating Curves (ROC) and % Area under the ROC curve (AUC) of the association between malnutrition risk and swallowing indices after adjusting for covariates. 1a) Self-reported swallowing problem (T-SSQ), and 1b) Objective tongue strength (18-kPa cut-off value).

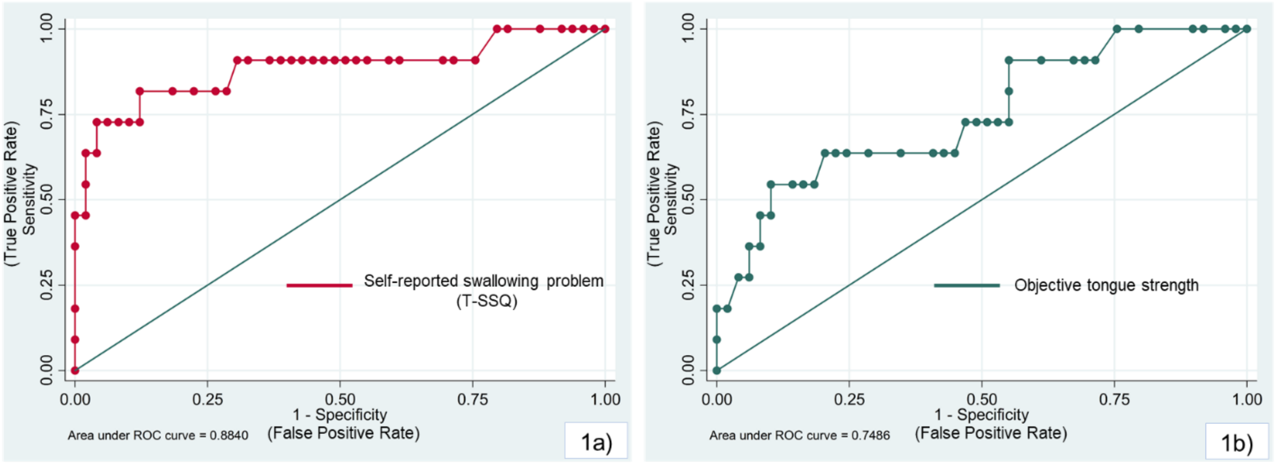

Estimates of the PPV, NPV, sensitivity, and specificity are presented in Table 3. The sensitivity value indicated that 45.5% and 36.4% of older adults having malnutrition risk would have a swallowing problem and lower tongue strength, respectively. The PPV values indicated that the participants with a swallowing problem were 1.5–2 folds more likely to have malnutrition risk than those who had lower tongue strength.

Table 3. PPV, NPV, sensitivity, and specificity (%) between swallowing indices and malnutrition risk.

Impaired swallowing ability	Malnutrition risk: (%)			
	PPV	NPV	Sensitivity	Specificity
Self-reported swallowing problem by TSSQ (Yes)	71.4	87.2	45.5	96.0
Low tongue strength: (< 18 kPa)	40.0	86.0	36.4	87.8

Discussion

The present pilot study developed the Thai-version of the simplified swallowing questionnaire (T-SSQ) as a subjective index to evaluate the swallowing ability in older adults. The convergent validity of the T-SSQ was verified using objective tongue pressure as a reference. The findings from this pilot study revealed an association between the subjective and objective swallowing indices and malnutrition risk. Sensitivity analysis demonstrated that the ability of the T-SSQ in estimating malnutrition risk was better than objective tongue strength. In this study, the standard EAT-10 was not used as a subjective swallowing index because some of our patients were unable to understand and complete the EAT-10 questionnaire. Due to its complexity and being time-consuming, our study introduced the T-SSQ for evaluating swallowing ability in older adults. The T-SSQ comprises only 4-item questions with a dichotomous answer, which is simpler than the 10-item questions answered using the 5-point Likert scale in the EAT-10.

Dependent status was significantly associated with low swallowing ability and malnutrition risk. Although the dependency level was associated with advanced age, a higher age was associated with lower tongue strength, but not having a swallowing problem. As supported by previous studies in healthy adults and older people, maximum tongue strength reduced with advanced age [20-22], which might be due to reduced musculoskeletal function [23] and masticatory muscle strength [20]. These findings imply that dependency status has a greater influence on swallowing ability than chronological age. Therefore, maintaining functional health and being active are important to prevent the progression of oral hypofunction in older adults.

The number of remaining teeth, posterior occlusal support, and denture type were not associated with the subjective or objective swallowing indices. Previous studies found that maximum tongue pressure increased with greater posterior occlusal support assessed using the Eicher index [20, 21]. In the present study, however, all edentulous patients wore a dental prosthesis when performing the tongue strength measurement because most of them required anterior denture teeth to position the pressure bulb. Wearing a dental prosthesis increases the number of posterior occlusal contacts, and therefore, enhances the bite force in edentulous individuals [24]. Individuals with higher occlusal forces present higher masticatory muscle strength [20], which is associated with lower dysphagia risk [15, 25]. Thus, wearing a dental prosthesis might reduce malnutrition risk in edentulous older adults regardless of the remaining functional teeth and posterior occlusal support.

In accordance with previous studies in middle-aged and older adults, malnutrition risk was associated with low tongue strength [3, 26]. To categorize low and high tongue strength, our study chose a cut-off value of 18 kPa because it gave the highest AUC value when plotting the ROC curve. The Japanese Society of Gerodontology suggests using 30 kPa as a cut-off value to diagnose decreased tongue strength [2], because a study in Japanese older adults reported that individuals with at least 30-kPa maximum tongue pressure could consume regular food [27]. Furthermore, a study in Canadian older adults in long-term care used a value of 26 kPa, the average tongue pressure of the study samples, as the cut-off value to categorize tongue pressure into low and high levels [3]. In our study, however, using either 30- or 26-kPa tongue pressure as a cut-off value gave relatively low sensitivity and PPV in estimating malnutrition risk, and resulted in a nonsignificant association with a subjective swallowing problem determined by the T-SSQ. However, due to the limited sample size in this pilot study, the 18-cut off value cannot

yet be defined as representative of the Thai population. Because the thickness of the swallowing muscles might be different among ethnicities [28], individual studies may need to identify the ethnic-specific normal values of tongue strength.

The sensitivity of the subjective and objective swallowing indices in estimating malnutrition risk was comparable. However, the PPV value of the T-SSQ was about 1.8-fold greater than that of tongue strength. Moreover, the AUC obtained from the T-SSQ and malnutrition risk was 15.2% higher than the objective tongue strength value. These findings indicated that the T-SSQ might be a more appropriate tool for estimating malnutrition risk in older adults. As supported by earlier studies [11, 29], recognizing signs and symptoms with a thorough history taking is key in early diagnosis and detecting swallowing impairment. Based on our results, we suggest that tongue strength measurement could be a supplemental tool to confirm the subjective finding whenever patients or their caregivers have communication problems or are unaware of the symptoms.

Treating oral frailty and oral hypofunction requires a multidisciplinary approach. Thus, dentists can be part of a holistic team by early detection of declined swallowing function to prevent the progression into the irreversible dysfunction stage [2]. The present study suggests using the T-SSQ as a screening method for evaluating the swallowing ability in older adults that does not require an experienced physician in routine dental practice. In addition, we propose a concept for identifying a cut-off value to categorize lower and higher tongue strength using malnutrition risk as an outcome.

Some limitations were noted in this study. Because all edentulous older adults in this study wore a dental prosthesis and visited the dental clinic for maintenance recall, the swallowing ability of the edentulous patients without a denture or with an ill-fitting denture was

not evaluated. The present pilot study did not include patients who were diagnosed with dysphagia by physicians as a positive control because we wanted to develop a screening tool for swallowing impairment rather than a tool for dysphagia diagnosis. Due to the limited number of samples with 70% study power, the cut-off tongue strength value cannot yet be generalized to other populations. Further study in a larger population is required to verify the reliability, validity, and sensitivity of the questionnaire. Measurement equivalence of the T-SSQ should be performed in older adults with different cultural backgrounds. Convergent validity should be done with other standard subjective swallowing indexes, such as EAT-10 and objective tools, such as video fluoroscopy and fiberoptic endoscopic examination of swallowing [2, 30, 31]. Further use of the simplified questionnaire for early detection of swallowing problem in a clinic and community-based study by caregiver and non-healthcare personnel should be evaluated.

Conclusions

Self-reported swallowing problems determined by the T-SSQ were associated with objective tongue strength, indicating convergent validity of the newly developed subjective index. Both subjective T-SSQ and objective tongue strength indices were associated with malnutrition risk in older adults. However, the subjective T-SSQ better estimated malnutrition risk than the objective tongue strength.

Acknowledgments

The authors gratefully acknowledge Dr. Kevin Tompkins for language revision of the manuscript.

Funding

This research was funded by Chulalongkorn University, Grant number CU_GR_63_11_32_04. The funders had no role in study design, data collection and analysis, decision to publish, or preparation of the manuscript

Conflict of interest

The authors declare no potential conflict of interest and no competing interest in this study

Data availability statement

All relevant data are within the paper and its supporting information files.

References

1. Razak, P. A.; Richard, K. M.; Thankachan, R. P.; Hafiz, K. A.; Kumar, K. N.;Sameer, K. M. Geriatric oral health: a review article. *J Int Oral Health* **2014**,6(6),110-116.
2. Minakuchi, S.; Tsuga, K.; Ikebe, K.; Ueda, T.; Tamura, F.; Nagao, K., et al. Oral hypofunction in the older population: Position paper of the Japanese Society of Gerodontology in 2016. *Gerodontology* **2018**,35(4),317-324.
3. Namasivayam-MacDonald, A. M.; Morrison, J. M.; Steele, C. M.;Keller, H. How Swallow Pressures and Dysphagia Affect Malnutrition and Mealtime Outcomes in Long-Term Care. *Dysphagia* **2017**,32(6),785-796.
4. Sura, L.; Madhavan, A.; Carnaby, G.;Crary, M. A. Dysphagia in the elderly: management and nutritional considerations. *Clin Interv Aging* **2012**,7(287-298.

5. Rasheed, S.;Woods, R. T. Malnutrition and quality of life in older people: a systematic review and meta-analysis. *Ageing Res Rev* **2013**,12(2),561-566.

6. Clark, H. M.; Henson, P. A.; Barber, W. D.; Stierwalt, J. A.;Sherrill, M. Relationships among subjective and objective measures of tongue strength and oral phase swallowing impairments. *Am J Speech Lang Pathol* **2003**,12(1),40-50.

7. Chatindiara, I.; Allen, J.; Popman, A.; Patel, D.; Richter, M.; Kruger, M.;Wham, C. Dysphagia risk, low muscle strength and poor cognition predict malnutrition risk in older adults athospital admission. *BMC Geriatr* **2018**,18(1),78.

8. Hansen, T.;Kjaersgaard, A. Item analysis of the Eating Assessment Tool (EAT-10) by the Rasch model: a secondary analysis of cross-sectional survey data obtained among community-dwelling elders. *Health Qual Life Outcomes* **2020**,18(1),139.

9. Wilmskoetter, J.; Bonilha, H.; Hong, I.; Hazelwood, R. J.; Martin-Harris, B.;Velozo, C. Construct validity of the Eating Assessment Tool (EAT-10). *Disabil Rehabil* **2019**,41(5),549-559.

10. Walker HK; Hall WD; Hurst JW;editors: **Chapter 82 - Dysphagia. Clinical Methods: The History, Physical, and Laboratory Examinations. 3rd edition** Boston: Butterworths; 1990.

11. Nawaz, S.;Tulunay-Ugur, O. E. Dysphagia in the Older Patient. *Otolaryngol Clin North Am* **2018**,51(4),769-777.

12. WHO: **World Health Organization. Process of translation and adaptation of instruments**. In.; 2013.

13. Panutat S, C. C., Nuntawan C, Pumsrisawat A, Pruksacheva T. Caring for the elderly with malnutrition. *Journal of Rachathani Innovation Health Science* **2017**,1(1),1-15.

14. Handbook for standard and operation of qualified elderly clinic. In.: Institute of Geriatric Medicine, Department of Medical Service, Ministry of Public Health 2013.

15. Hara, K.; Tohara, H.; Wada, S.; Iida, T.; Ueda, K.;Ansai, T. Jaw-opening force test to screen for Dysphagia: preliminary results. *Arch Phys Med Rehabil* **2014**,95(5),867-874.

16. Machida, N.; Tohara, H.; Hara, K.; Kumakura, A.; Wakasugi, Y.; Nakane, A.;Minakuchi, S. Effects of aging and sarcopenia on tongue pressure and jaw-opening force. *Geriatr Gerontol Int* **2017**,17(2),295-301.

17. Hayashi, R.; Tsuga, K.; Hosokawa, R.; Yoshida, M.; Sato, Y.;Akagawa, Y. A novel handy probe for tongue pressure measurement. *Int J Prosthodont* **2002**,15(4),385-388.

18. Rockwood, K.; Song, X.; MacKnight, C.; Bergman, H.; Hogan, D. B.; McDowell, I.;Mitnitski, A. A global clinical measure of fitness and frailty in elderly people. *CMAJ* **2005**,173(5),489-495.

19. Thai Cognitive Test Development Committee 1999. Mini-Mental State Examination-Thai 2002 Bangkok: Institute of Geriatric Medicine, Department of Medical Services, Ministry of Public Health, Thailand; 2002.

20. Hara, K.; Tohara, H.; Kenichiro, K.; Yamaguchi, K.; Ariya, C.; Yoshimi, K.; Nakane, A.;Minakuchi, S. Association between tongue muscle strength and masticatory muscle strength. *J Oral Rehabil* **2019**,46(2),134-139.

21. Kikutani, T.; Tamura, F.; Nishiwaki, K.; Kodama, M.; Suda, M.; Fukui, T., et al. Oral motor function and masticatory performance in the community-dwelling elderly. *Odontology* **2009**,97(1),38-42.

22. Higa, C.; Mori, T.; Hiraoka, A.; Takeda, C.; Kuroki, A.; Yoshikawa, M.; Yoshida, M.;Tsuga, K. Five-year change in maximum tongue pressure and physical function in community-dwelling elderly adults. *J Dent Sci* **2020**,15(3),265-269.

23. Kletzien, H.; Cullins, M. J.;Connor, N. P. Age-related alterations in swallowing biomechanics. *Exp Gerontol* **2019**,118(45-50.

24. Limpuangthip, N.; Somkotra, T.;Arksornnukit, M. Subjective and objective measures for evaluating masticatory ability and associating factors of complete denture wearers: A clinical study. *J Prosthet Dent* **2021**,125(2),287-293.

25. Maeda, K.;Akagi, J. Decreased tongue pressure is associated with sarcopenia and sarcopenic dysphagia in the elderly. *Dysphagia* **2015**,30(1),80-87.

26. Nakazawa, Y.; Kikutani, T.; Igarashi, K.; Yajima, Y.;Tamura, F. Associations between tongue strength and skeletal muscle mass under dysphagia rehabilitation for geriatric out patients. *J Prosthodont Res* **2020**,64(2),188-192.

27. Tanaka Y, N. Y., Yokoo M, Takeda Y, Yamada K, Kayashita J. Examination about the relation of meal form, tongue pressure, grip and walking state in inpatient and elderly residents. *Jpn J Dysphagia Rehabil* **2015**,19(52-62.

28. Jensen, B.; Moritoyo, T.; Kaufer-Horwitz, M.; Peine, S.; Norman, K.; Maisch, M. J., et al. Ethnic differences in fat and muscle mass and their implication for interpretation of bioelectrical impedance vector analysis. *Appl Physiol Nutr Metab* **2019**,44(6),619-626.

29. Malagelada, J.; Bazzoli, F.; Boeckxstaens, G.; De Looze, D.; Fried, M.; Kahrilas, P., et al. Dysphagia: Global Guidelines and Cascades. *World Gastroenterology Organisation* **2014**

30. Nishiwaki, K.; Tsuji, T.; Liu, M.; Hase, K.; Tanaka, N.;Fujiwara, T. Identification of a simple screening tool for dysphagia in patients with stroke using factor analysis of multiple dysphagia variables. *J Rehabil Med* **2005**,37(4),247-251.

31. Lim, S. H.; Lieu, P. K.; Phua, S. Y.; Seshadri, R.; Venketasubramanian, N.; Lee, S. H.;Choo, P. W. Accuracy of bedside clinical methods compared with fiberoptic endoscopic examination of swallowing (FEES) in determining the risk of aspiration in acute stroke patients. *Dysphagia* **2001**,16(1),1-6.

Figure

Click here to access/download;Figure;Fig1_Swallow_revise1.docx

Figure 1. Receiver Operating Curves (ROC) and % Area under the ROC curve (AUC) of the association between malnutrition risk and swallowing indices after adjusting for covariates. 1a) Self-reported swallowing problem (T-SSQ), and 1b) Objective tongue strength (18-kPa cut-off value).

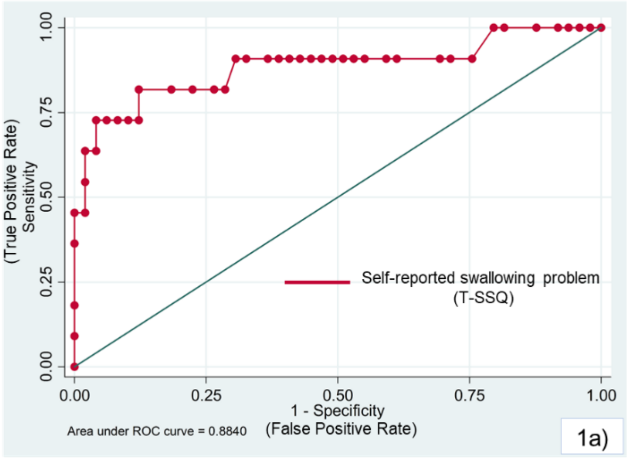

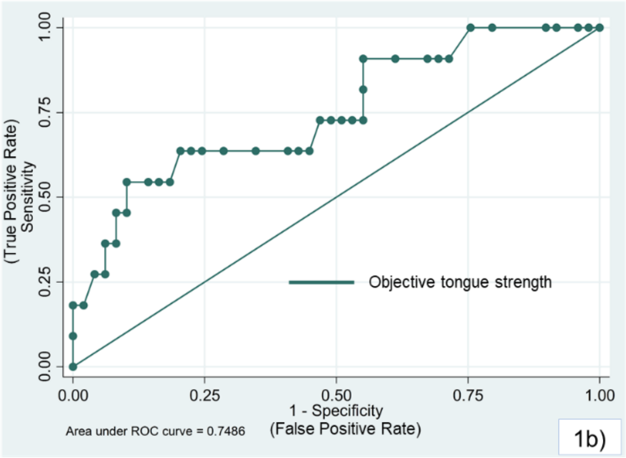

1b)

Supporting Information1

Click here to access/download

Supporting Information

Supplementary File1_Table_swallow.docx

Click here to access/download
Supporting Information
Supplement File2_Questionnaire Thai_Eng.docx

Raw data

Click here to access/download
Supporting Information
Swallow_PlosOne.xlsx

Title page

A simplified method for evaluating swallowing ability and estimating malnutrition risk ~~in older adults~~: A pilot study in older adults

Short title: Swallowing ability and malnutrition risk

Nareudee Limpuangthip[1¶], Orapin Komin[1¶*], Teerawut Tatiyapongpaiboon[2]

[1]Department of Prosthodontics, Faculty of Dentistry, Chulalongkorn University, Bangkok, Thailand.

[2]Thungyai Hospital, Nakhon Si Thammarat, Thailand

[¶]These authors equally contributed to this study

*** Corresponding author:**

E-mail: Orapin.geriatric@gmail.com

Abstract

Objectives: The aim of this pilot study was to develop a Thai-version of a simple swallowing questionnaire, called the T-SSQ, and ~~T~~to evaluate the association between malnutrition risk and swallowing ability, determined objectively by tongue strength and subjectively by the T-SSQ~~a newly-developed 4-item questionnaire~~. Sensitivity analysis was also performed to determine which swallowing indices better estimate malnutrition in older adults.

Methods: This cross-sectional study comprised two phases: Phase I, development and cross-cultural translation of the T-SSQ; and Phase II, application of the T-SSQ in~~included~~ 60 older adults. In Phase I, content and face validity of the T-SSQ was evaluated by 10 experts and 15 older adults. In Phase II, the convergent validity of the T-SSQ was evaluated by determining its association with objective tongue strength. ~~The dependent variable was n~~Nutritional status was evaluated using the Thai-version of the Mini-Nutritional Assessment. Covariates included sociodemographic characteristics, and oral and health-related status. ~~The independent variables were subjective and objective swallowing ability, evaluated using a 4-item questionnaire of swallowing problems and tongue strength, respectively.~~ Adjusting for covariates, the associations between the two swallowing indices and malnutrition risk were determined using multivariable regression analyses. A cut-off value for low tongue strength was determined using a receiver operating characteristic (ROC) curve, and sensitivity analysis between the swallowing indices and malnutrition risk was performed.

Results: The T-SSQ comprised 4-items of common signs and symptoms of a swallowing problem. Its content and face validity were verified. Older adults were considered as having a swallowing problem when at least one item was reported. Convergent validity of the subjective

index was revealed shown by significantly different tongue strength values between the participants with and without a swallowing problem (p for independent t-test = 0.014).by its significant association with objective tongue strength. Based on the highest area under the ROC curve, an 18-kPa cut-off value was chosen to classify low tongue strength. Having a swallowing problem and low tongue strength was significantly associated with malnutrition risk. The positive predictive value PPV values of the subjective swallowing index was about 1.8-fold higher than objective tongue strength.

Conclusions: Self-reported swallowing problems determined by the T-SSQ can be used as a subjective index for evaluating swallowing ability in older adults. Subjective swallowing problems and objective tongue strength were associated with malnutrition risk. However, the subjective index T-SSQ better estimated malnutrition risk better than the objective index.

Keywords: Nutritional assessment, Oral function, Swallowing function, Tongue function, Tongue pressure.

Introduction

Oral and general health functionally decline as people age [1]. Gradually declined oral function can lead to oral frailty followed by oral hypofunction. However, they can recover to the healthy stage by early detection and proper dental treatment [2]. Oral health becomes oral frailty when a person has decrease[illegible]cluding teeth, increased unchewable foods, or slight choking/spillage while eating. Moreover, oral hypofunction is diagnosed when 3 out of 7 oral signs or symptoms are present: oral uncleanness and dryness, reduced occlusal force, reduce[illegible] chewing function, reduced tongue and lip motor function, and as well as reduced tongue pressure

and swallowing function [2]. Because eating and swallowing ability plays a major role in oral function, a decline in swallowing ability contributes to malnutrition [3, 4]. Malnutrition increases the risk of morbidity and mortality, and negatively affect the quality of life of older adults [5].

To prevent malnutrition in older adults, early detection of declined swallowing ability is necessary. Several objective and subjective indices have been used to evaluate swallowing ability in older adults. Tongue pressure measurement is commonly used to objectively evaluate swallowing ability, because tongue motor function plays an important role in mastication and swallowing [3, 6]. However, this method requires special instruments and time to perform. To evaluate swallowing ability subjectively, the 10-item Eating Assessment Tool (EAT-10) is commonly used because it is considered a reliable and validated questionnaire [2, 6, 7]. However, some studies reported the limitations of EAT-10 regarding its substantial floor effect, several redundant items, and relatively low construct validity [8, 9]. Thus, an alternative simple screening method for evaluating swallowing ability should be proposed for the early detection of oral function when a patient is in the frailty or hypofunction stage.

The aims of the present study werewas to develop a Thai-version of a simplified swallowing questionnaire (T-SSQ), and to evaluate the association between malnutrition risk and swallowing ability, determined objectively by tongue strength and subjectively by the T-SSQa newly-developed 4-item questionnaire. In addition, sensitivity analysis was performed to determine which swallowing indices better estimate malnutrition in older adults.

Materials and Methods

Study design and participants

The present study was a cross-sectional design. The study protocol was approved by the Ethics Committee of the Faculty of Dentistry, Chulalongkorn University (HREC-DCU 2018-112). The participants and their guardians providedgave written informed consent prior to participating in the study.

The participants were older adults (aged ≥ 60 years old) recruited from patients who received dental treatment at the Geriatric and Special Patients Care Clinic, Faculty of Dentistry, Chulalongkorn University during August 2017– January 2019. The exclusion criteria were patients who declined or were unable to perform a tongue pressure test due to severely declined functional or intellectual conditions, or currently had malnutrition. Based on these criteria, 61 older adults aged ≥ 60 years old participated in the study.

Power analysis of the sample size was calculated based on the null hypothesis of two independent proportions. The results indicated that the prevalence of malnutrition risk in participants who reported swallowing problem (n_1=7) and those who did not (n_2=54) were 0.71 and 0.11, respectively. Thus, a power of 95.2% was calculated at 5% type I error.

Phase I. Development of simplified swallowing questionnaire

A 9-item swallowing questionnaire (SQ) was created based on the common signs and symptoms of dysphagia patients according to Walker et al (1990) [10] and Nawaz S & Tulunay-Ugur OE (2018) [11] (Supplementary file 1). Cross-cultural translation of the SQ was performed according to the WHO guidelines [12]. A forward translation from English to the Thai version was conducted by two-independent translators, one dentist and one non-dentist, and integrated into a single Thai version. A back translation from Thai to English version was then conducted

by two-independent translators, one was a dentist and the other was not. To verity the content validity of the translated version, the principal investigators and translators discussed any discrepancies related to the meaning of words and phrases between the Thai and English versions. The investigators ensured that the basic concepts and meanings of all terms with reference to the original version were maintained, and then, the Thai-version of the swallowing questionnaire (T-SQ) was proposed.

The content validity of the T-SQ was evaluated by 10 experts (5 physicians and 5 dentists). They gave responses whether they agreed that each of the 9 items indicated a swallowing problem (agree, disagree). The items with less than 80% agreement were excluded, and the final version was reduced to 4 items. A face validity of the 4-item swallowing questionnaire was evaluated in 20 older adults (age range 52–77 years). They gave responses whether they clearly understood the description of each item using a dichotomous scale (clearly understood and unclear), and provided suggestions if any rephrasing was necessary for easier understanding. All items received 90–100% clearly understood response but with some suggestions. After the discrepancies were discussed, a final version of the Thai-version simplified swallowing questionnaire (T-SSQ) was developed. The word liquid in the English version was translated into naam in the Thai version, which means water (noun) or related to liquid substance (adjective) for better understanding by Thais.

The T-SSQ comprised 4 items: 1) having problems swallowing certain food or liquids, or could not swallow at all, 2) coughing or choking when eating or drinking, 3) aspirations with liquids or solid food occurs, and 4) a sensation that food got stuck in the throat or chest. The response to each item was given as having (1) or not having (0) a problem. A person was defined

as having a swallowing problem when at least one of items was present at least once a week during the past month.

Phase II. Application of the T-SSQ

Phase II, the T-SSQ application, was conducted as a pilot study in 60 older adults who did not participate in Phase I. Swallowing ability was assessed subjectively by the T-SSQ and objectively by tongue strength. Convergent validity of the T-SSQ was evaluated using objective tongue pressure as a reference. Sensitivity analysis was performed to determine which subjective and objective swallowing indices better estimate malnutrition in older adults.

Nutritional assessmentDependent variables

Nutritional status was measured using the Thai-version of the Mini-Nutritional Assessment (MNA) with a score ranging from 0–30. The participants were categorized as having malnutrition risk when MNA score = 17–23.5, and being normal when the score ≥24. The MNA was used because it is a standardized and validated instrument in older adults [13, 14].

Independent variablesSwallowing ability assessment

In this study, the subjective swallowing index was a 4-item questionnaire, in which the items were adopted from common signs and symptoms reported by patients with swallowing problems [11].

Subjective Sswallowing ability was evaluated using the T-SSQ developed in Phase I. The participants were interviewed by interviewing the participants with the assistance of their caregivers, if present. The participants were defined as having a swallowing problem when at least one of the following signs and symptoms was present at least once a week within the past

~~month: 1) having problems swallowing certain food or liquids, or could not swallow at all, 2) coughing or choking when eating or drinking, 3) aspirations with liquids or solid food occurs, or 4) a sensation that food got stuck in the throat or chest.~~ The participants were defined as having a swallowing problem when at least one of the T-SSQ items was reported. The inter-examiner reliability was determined using 15 patients at the ~~first~~ patients' first evaluation visit. The ~~intra-examiner~~ test-retest reliability was evaluated by reinterviewing the~~se~~ 15 patients one week later. The weighted Kappa scores for the ~~inter- and intra~~inter-examiner and test-retest reliability assessments were approximately 0.82 and 0.87, respectively.

The objective swallowing ability was evaluated through tongue pressure, measured using the JMS TPM-02 measurement device (JMS, Inc., Hiroshima, Japan), which consisted of a plastic catheter and a balloon [15-17]. The participants sat in an upright position. The balloon was inserted into their oral cavity and placed on the anterior part of the palate with their lips and jaw closed, while the plastic catheter was held at the midpoint of the central incisors. The participants raised their tongue and pressed the balloon against the hard palate as hard as possible, and the maximum tongue pressure (kPa, kilopascal) was read. This procedure was done in triplicate with 5 min resting intervals, and the tongue strength (kPa) was calculated from the average value of the three measurements [15-17]. ~~The objective tongue strength was used as a reference to assess the convergent validity of the proposed subjective swallowing index.~~

Covariates

Formatted: Indent: First line: 0.5"

Information regarding biological factors, oral- and health-related status was recorded. Biological factors were an individuals' age and sex. A ~~dental professional~~ dentist evaluated oral status, comprising the number of remaining functional teeth (ranged from 0 to 28 teeth), number

of posterior occluding pairs (ranged ~~from~~ 0–~~to~~ 8 pairs), and type of denture worn. If more than one type of denture was present~~ed~~, it was classified as the type with the~~a~~ higher number of tooth loss.

Health-related status covered the participants physical and psychological conditions: dependency status and cognitive status, respectively. The clinical frailty scale (CFS) was used to categorize dependency status into independent, semi-dependent, and dependent [18]. Cognitive function was evaluated using the Thai-version of the Mini-Mental State Evaluation (MMSE) [19]. With a score ranging from 0–30, the participants were considered as having mild cognitive impairment (MCI) when the score was below 18 and 22 when their educations was at least primary and above primary level, respectively [19].

Power analysis

A power analysis of the sample size was performed to determine whether the T-SSQ was a sensitive assessment tool for using as a key instrument in early diagnosis and detecting swallowing impairment among older adults. The results indicated that the tongue strength (mean ±s.d.) of the participants who reported a swallowing problem (n_1=7) and those who did not (n_2=54) were 16.8 ±10.6 kPa and 26.7 ±9.6 kPa, respectively. Using the two-independent means test, a power of 70% was calculated at 5% type I error.

Data analysis

Descriptive statistics was performed to determine the percentage (%) and mean ±standard deviation (s.d.). Univariate analyses of the associations between related variables and having a swallowing problem were analyzed using the chi-squared test, whereas its association with tongue strength and MNA score were analyzed using either one-way ANOVA or independent t-

test. Variables with p-value < 0.10 were included in the multivariable analyses. Adjusting for covariates, multivariable logistic and linear regression were used to determine the factors associated with the subjective and objective swallowing indices, and their associations with malnutrition risk. To determine the convergent validity, the tongue strength values of the participants with and without swallowing problem assessed by the T-SSQ were compared using the independent t-test. A receiver operating characteristic (ROC) curve was plotted to determine the area under the curve (AUC) in the malnutrition risk models; the higher the AUC, the better the model was able to distinguish between the participants with and without malnutrition risk. To categorize the low and high tongue strength, a cut-off value that gave the highest AUC value was chosen. For the sensitivity analysis, the positive predictive value (PPV), negative predictive value (NPV), sensitivity, and specificity between malnutrition risk and the two swallowing ability indices were calculated. The data were analyzed using STATA version 13.0 (StataCorp LP) at a 5% significance level.

Results

The T-SSQ was developed as a subjective swallowing index, comprising 4-items of common signs and symptoms of a swallowing problem. The content and face validity of the T-SSQ was assessed. The characteristics of the participants attending in Phase II based on the subjective and objective swallowing indices, and malnutrition risk are shown in Table 1. Their mean ±s.d. age ~~of participants~~ was 78.0 ±7.0 years old. Malnutrition risk was found in 18% of the participants, while the others were within normal limits. MCI was present in 90% of the semi- and dependent older participants. Oral status was associated with the subjective and

objective swallowing indices, and malnutrition risk. The convergent validity of the T-SSQ ~~subjective swallowing problem~~ was revealed as shown by a significant difference in tongue strength values between the participants with and without a swallowing problem (p for independent t-test = 0.014).~~by its significant association with objective tongue strength .~~

Table 1. Characteristics of the study participants.

Variables	Overall distribution: %	Swallowing ability index: Self-reported swallowing problem by T-SSQ (Yes): %	Swallowing ability index: Maximum tongue pressure (kPa): mean (±s.d.)	Nutritional status: MNA score: mean (±s.d.)
Overall	***100.0***	***11.7***	***25.5 (±10.1)***	***26.0 (±3.0)***
Age (years): 60 – 69	15.0	11.1	34.5 (±8.7)	25.0 (±3.8)
70 – 79	40.0	4.2	26.8 (±9.3)*	26.5 (±2.1)
80 and above	45.0	18.5†	21.4 (±9.4)*	26.0 (±3.3)
Sex: Male	48.3	10.3	27.7 (±9.8)	26.9 (±2.1)
Female	51.7	12.9	23.5 (±10.1)†	25.2 (±3.4)
**Health-related status**				
Dependency status: Independent	71.7	4.6	27.8 (±9.8)	26.9 (±2.1)
Semi-dependent	18.3	27.3*	19.1 (±8.9)*	25.1 (±2.5)
Dependent	10.0	33.3*	21.2 (±8.8)*	21.1 (±3.9)*
MMSE score: Normal	78.3	6.2	26.5 (±10.4)	26.5 (±2.2)
Mild cognitive impairment	21.7	30.8*	22.1 (±8.6)†	24.1 (±4.4)
**Oral status**				
Natural teeth: 0	19.7	16.7	24.1 (±10.2)	26.0 (±3.6)
1–19	70.5	11.6	25.2 (±9.7)	25.9 (±3.0)
20 and above	9.8	0.0	30.1 (±9.9)	27.0 (±2.0)
Posterior occluding pairs: 0	76.7	13.0	24.5 (±10.2)	26.1 (±3.0)
1–3	13.3	12.5	32.4 (±10.7)	25.9 (±1.8)
4 and above	10.0	0.0	26.7 (±9.6)	25.4 (±4.5)
Denture type: No denture	3.3	0.0	30.7 (±1.8)	29.2 (±1.1)
Removable partial denture	45.0	7.4	27.3 (±8.8)	26.0 (±2.7)
Complete denture	51.7	16.1	23.6 (±11.1)	25.8 (±3.2)
**Swallowing ability**				
Having swallowing problem: No	11.7	-	26.7 (±9.6)	26.0 (±2.8)
Yes	88.3		16.8 (±10.6)*	23.0 (±2.7)*

*p <0.05, †p<0.10 determined by independent t-test or one-way ANOVA and post-hoc comparison test. ~~N/A, not applicable due to multicollinearity with dependency status.~~
~~OR, odds ratio; β, beta-coefficient; CI, confidence interval; ref, reference~~.

There were significant associations between the subjective and objective swallowing indices and malnutrition risk after adjusting for potential covariates (Table 2). Because there was

collinearity between dependency status and MCI, the MCI variable was not included in the multivariable regression models. Based on the ROC curve, 18 kPa was chosen as a cut-off value to categorize the participants into low and high tongue strength because it gave the highest AUC value when plotting the curve between tongue strength and malnutrition risk (Figure 1).

Table 2. The associations between swallowing indices and related variables.

Variables	Swallowing ability index		Malnutrition risk: adjusted OR (95% CI)	
	Self-reported swallowing problem by T-SSQ (Yes): adjusted OR (95% CI)	Maximum tongue pressure (kPa): adjusted β (95% CI)	Model 1	Model 2
Age (years old)	1.05 (0.92, 1.18)	-0.65 (-1.00, -0.30)*	1 (ref)	0.98 (0.84, 1.14)
Sex (Female)	0.55 (0.07, 4.38)	-1.00 (-5.76, 3.76)	6.28 (0.44, 45.4)	3.23 (0.45, 23.4)
Dependency status: Independent	1 (ref)	1 (ref)	1 (ref)	1 (ref)
Semi-dependent	8.44 (0.89.30.1)†	-4.19 (-10.6, 2.28)	0.21 (0.01, 6.26)	0.80 (0.07, 8.65)
Dependent	13.4 (1.24, 39.4)*	-6.67 (-14.1, 1.18)†	22.3 (1.53, 53.3)*	40.6 (3.30, 85.3)*
**Swallowing ability measures**				
Self-reported swallowing problem (Yes)			35.5 (3.49, 75.5)*	-
Maximum tongue pressure (<18 kPa)			-	0.11 (0.01, 0.71)*
AUC (%)			***88.3***	***74.9***

*p <0.05, †p<0.10. OR, odds ratio; β, beta-coefficient; CI, confidence interval; ref, reference; AUC, Area under the Receiverrd Operating CurveCharacteristic (ROC) curve.

Figure 1. Receiver Operating Curves (ROC) and % Area under the ROC curve (AUC) of the association between malnutrition risk and swallowing indices after adjusting for covariates. 1a) Self-reported ~~Subjective~~ swallowing problem (T-SSQ), and 1b) Objective tongue strength (18-kPa cut-off value).

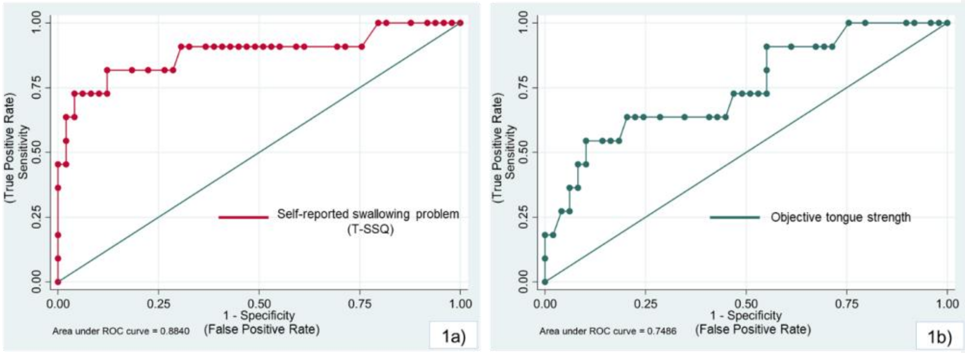

Estimates of the PPV, NPV, sensitivity, and specificity are presented in Table 3. The sensitivity value indicated that 45.5% and 36.4% of older adults having malnutrition risk would have a swallowing problem and lower tongue strength, respectively. The PPV values indicated that the participants with a swallowing problem were 1.5–2 folds more likely to have malnutrition risk than those who had lower tongue strength.

Table 3. PPV, NPV, sensitivity, and specificity (%) between swallowing indices and malnutrition risk.

Impaired swallowing ability	Malnutrition risk: (%)			
	PPV	NPV	Sensitivity	Specificity
Self-reported swallowing problem by TSSQ (Yes)	71.4	87.2	45.5	96.0
Low tongue strength: (< 18 kPa)	40.0	86.0	36.4	87.8

Discussion

The present pilot study developed the Thai-version of the simplified swallowing a 4-item questionnaire (T-SSQ) as a subjective index to evaluate the swallowing ability in older adults. The convergent validity of the subjective swallowing indexT-SSQ was verified using objective tongue pressure as a reference. The findings from this pilot study revealed anthe association between the subjective and objective swallowing indices and malnutrition risk. Sensitivity analysis demonstrated that the ability of the T-SSQsubjective swallowing index in estimating malnutrition risk was better than objective tongue strength. In this study, the standard EAT-10 was not used as a subjective swallowing index because some of our patients were unable to understand and complete the EAT-10 questionnaire. Due to its complexity and being time-consuming, our study introduced the T-SSQ a simplified questionnaire for evaluating swallowing ability in older adults. The T-SSQ questionnaire comprises only 4-item questions with a dichotomous answer, which is simpler than the 10-item questions answered using the 5-point Likert scale in the EAT-10.

Dependent status was significantly associated with low swallowing ability and malnutrition risk. Although the dependency level was associated with advanced age, a higher age was associated with lower tongue strength, but not having a swallowing problem. As supported by previous studies in healthy adults and older people, maximum tongue strength reduced with advanced age [20-22], which might be due to reduced musculoskeletal function [23] and masticatory muscle strength [20]. These findings imply that dependency status has a greater influence on swallowing ability than chronological age. Therefore, maintaining functional health and being active are important to prevent the progression of oral hypofunction in older adults.

The number of remaining teeth, posterior occlusal support, and denture type were not associated with the subjective or objective swallowing indices. Previous studies found that maximum tongue pressure increased with greater posterior occlusal support assessed using the Eicher index [20, 21]. In the present study, however, all edentulous patients wore a dental prosthesis when performing the tongue strength measurement because most of them required anterior denture teeth to position the pressure bulb. Wearing a dental prosthesis increases the number of posterior occlusal contacts, and therefore, enhances the bite force in edentulous individuals [24]. Individuals with higher occlusal forces present higher masticatory muscle strength [20], which is associated with lower dysphagia risk [15, 25]. Thus, wearing a dental prosthesis might reduce malnutrition risk in edentulous older adults regardless of the remaining functional teeth and posterior occlusal support.

In accordance with previous studies in middle-aged and older adults, malnutrition risk was associated with low tongue strength [3, 26]. To categorize low and high tongue strength, our study chose a cut-off value of 18 kPa because it gave the highest AUC value when plotting the ROC curve. The Japanese Society of Gerodontology suggests using 30 kPa as a cut-off value to diagnose decreased tongue strength [2],. because a study in Japanese older adults reported that individuals with at least 30-kPa maximum tongue pressure could consume regular food [27]. Furthermore, a study in Canadian older adults in long-term care used a value of 26 kPa, the average tongue pressure of the study samples, as the cut-off value to categorize tongue pressure into low and high levels [3]. In our study, however, using either 30- or 26-kPa tongue pressure as a cut-off value gave relatively low sensitivity and PPV in estimating malnutrition risk, and resulted in a nonsignificant association with a subjective swallowing problem determined by the T-SSQ. However, due to the limited sample size in this pilot study, the 18-cut off value cannot

yet be defined as representative of the Thai population. Because the thickness of the swallowing muscles might be different among ethnicities [28], individual studies may need to identify the ethnic-specific normal values of tongue strength.

The sensitivity of the subjective and objective swallowing indices in estimating malnutrition risk was comparable. However, the PPV value of the T-SSQ subjective index was about 1.8-fold greater than that of tongue strength. Moreover, the AUC obtained from the subjective swallowing index T-SSQ and malnutrition risk was 15.2% higher than the objective tongue strength value. These findings indicated that the T-SSQsubjective swallowing index might be a more appropriate tool for estimating malnutrition risk in older adults. As supported by earlier studies [11, 29], recognizing signs and symptoms with a thorough history taking is key in early diagnosis and detecting swallowing impairment. Based on our results, we suggest that Ttongue strength measurement could be a supplemental tool to confirm the subjective finding whenever patients or their caregivers have communication problems or are unaware of the symptoms.

Treating oral frailty and oral hypofunction requires a multidisciplinary approach. Thus, dental professionals dentists can be part of a holistic team by early detection of declined swallowing function to prevent the progression into the irreversible dysfunction stage [2]. ThisThe present study suggests using the T-SSQ a simplified 4-item questionnaire as a screening method for evaluating the swallowing ability in older adults that does not require an experienced physician in routine dental practice. In addition, we propose a concept for identifying a cut-off value to categorize lower and higher tongue strength using malnutrition risk as an outcome.

Some limitations were noted in this study. Because all edentulous older adults in this study wore a dental prosthesis and visited the dental clinic for maintenance recall, the

swallowing ability of the edentulous patients without a denture or with an ill-fitting denture was not evaluated. The present pilot study did not include patients who were diagnosed with dysphagia by physicians as a positive control because we wanted to develop a screening tool for swallowing impairment rather than a tool for dysphagia diagnosis. Due to the limited number of samples with 70% study power, the cut-off tongue strength value cannot yet be generalized to other populations. However, fFurther study in a larger population is required to verify the reliability, and validity, and sensitivity of the questionnaire. Measurement equivalence of the T-SSQ should be performed in older adults with different cultural backgrounds. Convergent validity should be done with other standard subjective swallowing indexes, such as EAT-10 and objective tools, such as video fluoroscopy and fiberoptic endoscopic examination of swallowing [2, 30, 31]. Further use of the simplified questionnaire for early detection of swallowing problem in a clinic and community-based study by caregiver and non-healthcare personnel should be evaluated.

Conclusions

Self-reported swallowing problems determined by the T-SSQ were associated with objective tongue strength, indicating convergent validity of the newly developed subjective index. The 4-item self-reported swallowing problems questionnaire was developed as a subjective index for evaluating swallowing ability in older adults. The convergent validity of the subjective index was verified using objective tongue strength as a reference. Both subjective T-SSQ and objective tongue strength indices were associated with malnutrition risk in older adults. However, the subjective swallowing indexT-SSQ better estimated malnutrition risk than the objective tongue strength.

Acknowledgments

The authors gratefully acknowledge Dr. Kevin Tompkins for language revision of the manuscript.

Funding

This research was funded by Chulalongkorn University, Grant number CU_GR_63_11_32_04. The funders had no role in study design, data collection and analysis, decision to publish, or preparation of the manuscript

Conflict of interest

The authors declare no potential conflict of interest and no competing interest in this study

Data availability statement

All relevant data are within the paper and its supporting information files.

References

1. Razak PA, Richard KM, Thankachan RP, Hafiz KA, Kumar KN, Sameer KM. Geriatric oral health: a review article. J Int Oral Health 2014;6(6):110-116.

2. Minakuchi S, Tsuga K, Ikebe K, Ueda T, Tamura F, Nagao K, et al. Oral hypofunction in the older population: Position paper of the Japanese Society of Gerodontology in 2016. Gerodontology 2018;35(4):317-324.

3. Namasivayam-MacDonald AM, Morrison JM, Steele CM, Keller H. How Swallow Pressures and Dysphagia Affect Malnutrition and Mealtime Outcomes in Long-Term Care. Dysphagia 2017;32(6):785-796.

4. Sura L, Madhavan A, Carnaby G, Crary MA. Dysphagia in the elderly: management and nutritional considerations. Clin Interv Aging 2012;7(287-298.

5. Rasheed S, Woods RT. Malnutrition and quality of life in older people: a systematic review and meta-analysis. Ageing Res Rev 2013;12(2):561-566.

6. Clark HM, Henson PA, Barber WD, Stierwalt JA, Sherrill M. Relationships among subjective and objective measures of tongue strength and oral phase swallowing impairments. Am J Speech Lang Pathol 2003;12(1):40-50.

7. Chatindiara I, Allen J, Popman A, Patel D, Richter M, Kruger M, Wham C. Dysphagia risk, low muscle strength and poor cognition predict malnutrition risk in older adults athospital admission. BMC Geriatr 2018;18(1):78.

8. Hansen T, Kjaersgaard A. Item analysis of the Eating Assessment Tool (EAT-10) by the Rasch model: a secondary analysis of cross-sectional survey data obtained among community-dwelling elders. Health Qual Life Outcomes 2020;18(1):139.

9. Wilmskoetter J, Bonilha H, Hong I, Hazelwood RJ, Martin-Harris B, Velozo C. Construct validity of the Eating Assessment Tool (EAT-10). Disabil Rehabil 2019;41(5):549-559.

10. Walker HK, Hall WD, Hurst JW, editors: Chapter 82 - Dysphagia. Clinical Methods: The History, Physical, and Laboratory Examinations. 3rd edition Boston: Butterworths; 1990.

11. Nawaz S, Tulunay-Ugur OE. Dysphagia in the Older Patient. Otolaryngol Clin North Am 2018;51(4):769-777.

12. WHO: World Health Organization. Process of translation and adaptation of instruments. In.; 2013.

13. Panutat S CC, Nuntawan C, Pumsrisawat A, Pruksacheva T. Caring for the elderly with malnutrition. Journal of Rachathani Innovation Health Science 2017;1(1):1-15.

14. Handbook for standard and operation of qualified elderly clinic. In.: Institute of Geriatric Medicine, Department of Medical Service, Ministry of Public Health 2013.

15. Hara K, Tohara H, Wada S, Iida T, Ueda K, Ansai T. Jaw-opening force test to screen for Dysphagia: preliminary results. Arch Phys Med Rehabil 2014;95(5):867-874.

16. Machida N, Tohara H, Hara K, Kumakura A, Wakasugi Y, Nakane A, Minakuchi S. Effects of aging and sarcopenia on tongue pressure and jaw-opening force. Geriatr Gerontol Int 2017;17(2):295-301.

17. Hayashi R, Tsuga K, Hosokawa R, Yoshida M, Sato Y, Akagawa Y. A novel handy probe for tongue pressure measurement. Int J Prosthodont 2002;15(4):385-388.

18. Rockwood K, Song X, MacKnight C, Bergman H, Hogan DB, McDowell I, Mitnitski A. A global clinical measure of fitness and frailty in elderly people. CMAJ 2005;173(5):489-495.

19. Thai Cognitive Test Development Committee 1999. Mini-Mental State Examination-Thai 2002 Bangkok: Institute of Geriatric Medicine, Department of Medical Services, Ministry of Public Health, Thailand; 2002.

20. Hara K, Tohara H, Kenichiro K, Yamaguchi K, Ariya C, Yoshimi K, Nakane A, Minakuchi S. Association between tongue muscle strength and masticatory muscle strength. J Oral Rehabil 2019;46(2):134-139.

21. Kikutani T, Tamura F, Nishiwaki K, Kodama M, Suda M, Fukui T, et al. Oral motor function and masticatory performance in the community-dwelling elderly. Odontology 2009;97(1):38-42.

22. Higa C, Mori T, Hiraoka A, Takeda C, Kuroki A, Yoshikawa M, Yoshida M, Tsuga K. Five-year change in maximum tongue pressure and physical function in community-dwelling elderly adults. J Dent Sci 2020;15(3):265-269.

23. Kletzien H, Cullins MJ, Connor NP. Age-related alterations in swallowing biomechanics. Exp Gerontol 2019;118(45-50.

24. Limpuangthip N, Somkotra T, Arksornnukit M. Subjective and objective measures for evaluating masticatory ability and associating factors of complete denture wearers: A clinical study. J Prosthet Dent 2021;125(2):287-293.

25. Maeda K, Akagi J. Decreased tongue pressure is associated with sarcopenia and sarcopenic dysphagia in the elderly. Dysphagia 2015;30(1):80-87.

26. Nakazawa Y, Kikutani T, Igarashi K, Yajima Y, Tamura F. Associations between tongue strength and skeletal muscle mass under dysphagia rehabilitation for geriatric out patients. J Prosthodont Res 2020;64(2):188-192.

27. Tanaka Y NY, Yokoo M, Takeda Y, Yamada K, Kayashita J. Examination about the relation of meal form, tongue pressure, grip and walking state in inpatient and elderly residents. Jpn J Dysphagia Rehabil 2015;19(52-62.

28. Jensen B, Moritoyo T, Kaufer-Horwitz M, Peine S, Norman K, Maisch MJ, et al. Ethnic differences in fat and muscle mass and their implication for interpretation of bioelectrical impedance vector analysis. Appl Physiol Nutr Metab 2019;44(6):619-626.

29. Malagelada J, Bazzoli F, Boeckxstaens G, De Looze D, Fried M, Kahrilas P, et al. Dysphagia: Global Guidelines and Cascades. World Gastroenterology Organisation 2014.

30. Nishiwaki K, Tsuji T, Liu M, Hase K, Tanaka N, Fujiwara T. Identification of a simple screening tool for dysphagia in patients with stroke using factor analysis of multiple dysphagia variables. J Rehabil Med 2005;37(4):247-251.

31. Lim SH, Lieu PK, Phua SY, Seshadri R, Venketasubramanian N, Lee SH, Choo PW. Accuracy of bedside clinical methods compared with fiberoptic endoscopic examination of swallowing (FEES) in determining the risk of aspiration in acute stroke patients. Dysphagia 2001;16(1):1-6.

Response to reviewers

We are pleased to submit our revised manuscript Number: PONE-D-21-21718. The title has been modified to '**A simplified method for evaluating swallowing ability and estimating malnutrition risk: A pilot study in older adults**.' The newly developed questionnaire has been named 'Thai-version of Simplified Swallowing Questionnaire' or 'T-SSQ'. The requested revisions have been made in the manuscript in track changes, and our point-by-point responses are below.

Reviewer No. 1:

Reviewer point #1: The authors have embarked on a novel method to identify a screening tool for evaluating swallowing ability in older adults. The manuscript is well written and systematically presented.

However, the conclusions made from this study needs further justification for application on a larger population because its reliability and validity is yet unclear. Probably, this can be introduced as a pilot study to further analyze on the sensitivity of the proposed 4 items self-reported assessment tool.

Author response #1: Thank you for your comment. We have revised our study limitations, further study suggestions and conclusion. Due to a limitation, the study has been changed into a pilot study.

Reviewer point #2: Things that need further clarification include the selection of the items, independence of each item from the other, the scoring value adopted for this tool, the measurement equivalence of the tool, etc.

Author response #2: Development of the T-SSQ including the selection of the items, the scoring value adopted for the T-SSQ, its interpretation, and its validity testing have been clarified in the Materials and Methods section (Phase I subsection, Page 5-6). We did not use any statistical test to evaluate the independence of each item from the other because the items were selected by experts.

Reviewer point #3: The authors do not mention in which language the questionnaire was administered; if in the local language, any translation was done and cross-culturally validated.

Author response #3**:** Thai language was used for the swallowing questionnaire. The cross-cultural translation from English to Thai version was performed, and the descriptions have been added in the Materials and Methods section (Phase I subsection, Page 5)

Reviewer point #4: The cut off value for the Objective Swallowing Assessment was way below that reported in cited literature and in case that cut-off is raised there is a statistically significant variation between the Objective and Subjective Swallowing tools applied in this study.

Author response #4**:** The descriptions about the cut-off value have been revised in the Discussion section (Page 15-16) according to the reviewer's suggestion.

Reviewer point #5: The sample size in this study needs to be evaluated if is adequate to introduce a sensitive assessment tool that can be used as a key instrument in early diagnosis and detecting of swallowing impairment among older adults.

Author response #5**:** The power analysis of the sample size has been revised by evaluating whether the T-SSQ was a sensitive assessment tool for using as a key instrument in early diagnosis and detecting swallowing impairment among older adults. The revisions have been made in the Materials and Methods section (Power analysis subsection, Page 9)

Reviewer No. 2:

Reviewer point #1: Control group should have been included.

Author response #1: Our pilot study did not include patients who were diagnosed with dysphagia by physicians. Therefore, a positive control group was not present in this study. This was because we wanted to develop a screening tool for swallowing ability impairment rather than a tool for dysphagia diagnosis. This limitation has been added in the Discussion section (Page 17).

Reviewer point #2: Newly developed questionnaire should be validated. Statistics data should be furnished completely.

Author response #2: Descriptions about the validation of the newly developed questionnaire (T-SSQ) has been added in the Materials and Methods section (Phase I subsection). The statistical analysis has also been revised.

Reviewer point #3: Relevant figures with legends to be provided. Copy of questionnaire should be given.

Author response #3: The relevant figures with legends have been provided in the manuscript file. A copy of the questionnaire (in Thai, and English translation) has been provided as a supplementary file.

Additional responses to the comments in the attached Pdf files:

Materials and Methods section

- The references of the Thai-version of the Mini-Nutritional Assessment (MNA) has been revised.

- The references for the tongue pressure measurement and the Thai-version of Mini-Mental State Evaluation (MMSE) have been added.
- To evaluate the reliability of the T-SSQ in older adults, the inter-examiner reliability was examined in 15 patients at the patients' first evaluation visit. Test-retest reliability was evaluated by reinterviewing these patients one week later. The descriptions have been revised in the Swallowing ability assessment subsection (Page 8).

Results section

- The results for the descriptive statistics have been demonstrated in the Result section and Tables.
- The statistical tests have been added as a footnote of Table 1.

Discussion section

- The patients who were unable to perform a tongue pressure test due to severely declined functional or intellectual conditions were excluded. This exclusion criterion has been included in the Materials and Methods section (Page 5).
- The study limitations have been added according to the reviewers' suggestions.

Sincerely yours

Orapin Komin

Corresponding author
