# Supplementary material for: A simplified method for evaluating swallowing ability and estimating malnutrition risk: A pilot study in older adults
Source: PLoS One. 2022 Feb 16;17(2):e0263896. doi: 10.1371/journal.pone.0263896 (PMC8849596; doi:10.1371/journal.pone.0263896)
Supplement: Attachment — Submitted filename: Response reviewer_swallow_PlosOne.docx [file pone.0263896.s006.docx]

**Response to reviewers**

We are pleased to submit our revised manuscript Number: PONE-D-21-21718. The title has been modified to ‘**A simplified method for evaluating swallowing ability and estimating malnutrition risk: A pilot study in older adults**.’ The newly developed questionnaire has been named ‘Thai-version of Simplified Swallowing Questionnaire’ or ‘T-SSQ’. The requested revisions have been made in the manuscript in track changes, and our point-by-point responses are below.

**Reviewer No. 1:**

Reviewer point #1: The authors have embarked on a novel method to identify a screening tool for evaluating swallowing ability in older adults. The manuscript is well written and systematically presented.

However, the conclusions made from this study needs further justification for application on a larger population because its reliability and validity is yet unclear. Probably, this can be introduced as a pilot study to further analyze on the sensitivity of the proposed 4 items self-reported assessment tool.

Author response #1: Thank you for your comment. We have revised our study limitations, further study suggestions and conclusion. Due to a limitation, the study has been changed into a pilot study.

Reviewer point #2: Things that need further clarification include the selection of the items, independence of each item from the other, the scoring value adopted for this tool, the measurement equivalence of the tool, etc.

Author response #2: Development of the T-SSQ including the selection of the items, the scoring value adopted for the T-SSQ, its interpretation, and its validity testing have been clarified in the Materials and Methods section (Phase I subsection, Page 5-6). We did not use any statistical test to evaluate the independence of each item from the other because the items were selected by experts.

Reviewer point #3: The authors do not mention in which language the questionnaire was administered; if in the local language, any translation was done and cross-culturally validated.

Author response #3: Thai language was used for the swallowing questionnaire. The cross-cultural translation from English to Thai version was performed, and the descriptions have been added in the Materials and Methods section (Phase I subsection, Page 5)

Reviewer point #4: The cut off value for the Objective Swallowing Assessment was way below that reported in cited literature and in case that cut-off is raised there is a statistically significant variation between the Objective and Subjective Swallowing tools applied in this study.

Author response #4: The descriptions about the cut-off value have been revised in the Discussion section (Page 15-16) according to the reviewer’s suggestion.

Reviewer point #5: The sample size in this study needs to be evaluated if is adequate to introduce a sensitive assessment tool that can be used as a key instrument in early diagnosis and detecting of swallowing impairment among older adults.

Author response #5: The power analysis of the sample size has been revised by evaluating whether the T-SSQ was a sensitive assessment tool for using as a key instrument in early diagnosis and detecting swallowing impairment among older adults. The revisions have been made in the Materials and Methods section (Power analysis subsection, Page 9)

**Reviewer No. 2:**

Reviewer point #1: Control group should have been included.

Author response #1: Our pilot study did not include patients who were diagnosed with dysphagia by physicians. Therefore, a positive control group was not present in this study. This was because we wanted to develop a screening tool for swallowing ability impairment rather than a tool for dysphagia diagnosis. This limitation has been added in the Discussion section (Page 17).

Reviewer point #2: Newly developed questionnaire should be validated. Statistics data should be furnished completely.

Author response #2: Descriptions about the validation of the newly developed questionnaire (T-SSQ) has been added in the Materials and Methods section (Phase I subsection). The statistical analysis has also been revised.

Reviewer point #3: Relevant figures with legends to be provided. Copy of questionnaire should be given.

Author response #3: The relevant figures with legends have been provided in the manuscript file. A copy of the questionnaire (in Thai, and English translation) has been provided as a supplementary file.

**Additional responses to the comments in the attached Pdf files:**

**Materials and Methods section**

- The references of the Thai-version of the Mini-Nutritional Assessment (MNA) has been revised.
- The references for the tongue pressure measurement and the Thai-version of Mini-Mental State Evaluation (MMSE) have been added.
- To evaluate the reliability of the T-SSQ in older adults, the inter-examiner reliability was examined in 15 patients at the patients’ first evaluation visit. Test-retest reliability was evaluated by reinterviewing these patients one week later. The descriptions have been revised in the Swallowing ability assessment subsection (Page 8).

**Results section**

- The results for the descriptive statistics have been demonstrated in the Result section and Tables.
- The statistical tests have been added as a footnote of Table 1.

**Discussion section**

- The patients who were unable to perform a tongue pressure test due to severely declined functional or intellectual conditions were excluded. This exclusion criterion has been included in the Materials and Methods section (Page 5).
- The study limitations have been added according to the reviewers’ suggestions.

Sincerely yours

Orapin Komin

Corresponding author
