# Supplementary material for: A simplified method for evaluating swallowing ability and estimating malnutrition risk: A pilot study in older adults
Source: PLoS One. 2022 Feb 16;17(2):e0263896. doi: 10.1371/journal.pone.0263896 (PMC8849596; doi:10.1371/journal.pone.0263896)
Supplement: Attachment — Submitted filename: Response reviewer_swallow_PlosOne_revise2.docx [file pone.0263896.s008.docx]

**Response to reviewers**

We are pleased to submit our revised manuscript Number: PONE-D-21-21718R1, entitle ‘**A simplified method for evaluating swallowing ability and estimating malnutrition risk: A pilot study in older adults**.’ The requested revisions have been made in the manuscript in track changes, and our point-by-point responses are below.

**Reviewers:**

Reviewer point #1: Introduction section

Author response #1:

- The word ‘decreased occluding teeth’ has been revised to ‘decreased occluding pairs of natural teeth’
- The word ‘reduced chewing function’ has been revised to ‘declined masticatory function’

Reviewer point #2: Materials and Methods section.

Author response #2:

- Study design and participants subsection (Page 5): The sentence has been revised to ‘The participants were older adults (aged ≥ 60 years old) recruited from patients who received dental treatment at the Geriatric and Special Patients Care Clinic, Faculty of Dentistry, Chulalongkorn University during August 2017– January 2019 (total duration of 1 year and 5 months).’
- Covariates subsection (Page 8): The sentence has been revised to ‘With a score ranging from 0–30, the participants were considered as having mild cognitive impairment (MCI) when the score was below 18 and below 22 when they had attained at least primary and above primary education, respectively.’

Reviewer point #3: Discussion section

- Page 14: The sentence has been revised to ‘The T-SSQ comprises only 4-item questions with a dichotomous answer, which is simpler than the 10-item questions answered using the 5-point Likert scale in the standard EAT-10.’
- Page 16: The limitations of the study have been rephrased.

Sincerely yours

Orapin Komin

Corresponding author
